# Clinical Stratification of High-Grade Ovarian Serous Carcinoma Using a Panel of Six Biomarkers

**DOI:** 10.3390/jcm8030330

**Published:** 2019-03-08

**Authors:** Swapnil C. Kamble, Arijit Sen, Rahul D. Dhake, Aparna N. Joshi, Divya Midha, Sharmila A. Bapat

**Affiliations:** 1 National Centre for Cell Science, Savitribai Phule Pune University, Ganeshkhind, Pune, Maharashtra 411007, India; sckamble@unipune.ac.in; 2 Department of Technology, Savitribai Phule Pune University, Pune, Maharashtra 411007, India; 3 Department of Pathology, Armed Forces Medical College, Pune, Maharashtra 411040, India; aseniaf@gmail.com; 4 Department of Histopathology, Inlaks and Budrani Hospital, Morbai Naraindas Cancer Institute, Koregaon Park, Pune, Maharashtra 411001, India; drrahuldhake@gmail.com; 5 Department of Pathology, KEM Hospital, Pune, Maharashtra 411011, India; draparnajoshi@gmail.com; 6 Department of Oncopathology, Tata Medical Centre, Kolkata, West Bengal 700 156, India; divya.midha@tmckolkata.com

**Keywords:** high-grade serous ovarian carcinoma, epithelial-to-mesenchymal transition, molecular stratification, biomarkers, scoring system, immunohistochemistry

## Abstract

Molecular stratification of high-grade serous ovarian carcinoma (HGSC) for targeted therapy is a pertinent approach in improving prognosis of this highly heterogeneous disease. Enabling the same necessitates identification of class-specific biomarkers and their robust detection in the clinic. We have earlier resolved three discrete molecular HGSC classes associated with distinct functional behavior based on their gene expression patterns, biological networks, and pathways. An important difference revealed was that Class 1 is likely to exhibit cooperative cell migration (CCM), Class 2 undergoes epithelial to mesenchymal transition (EMT), while Class 3 is possibly capable of both modes of migration. In the present study, we define clinical stratification of HGSC tumors through the establishment of standard operating procedures for immunohistochemistry and histochemistry based detection of a panel of biomarkers including TCF21, E-cadherin, PARP1, Slug, AnnexinA2, and hyaluronan. Further development and application of scoring guidelines based on expression of this panel in cell line-derived xenografts, commercial tissue microarrays, and patient tumors led to definitive stratification of samples. Biomarker expression was observed to vary significantly between primary and metastatic tumors suggesting class switching during disease progression. Another interesting feature in the study was of enhanced CCM-marker expression in tumors following disease progression and chemotherapy. These stratification principles and the new information thus generated is the first step towards class-specific personalized therapies in the disease.

## 1. Introduction

Personalized therapeutic decisions in cancer necessitate the development of accurate stratification schemes based on mutations and/or association of tumor sub-groups with specific biomarkers and biological functions, besides well-elucidated principles for their detection [1]. Recent resolution of four gastric cancer molecular groups identified predictive amplifications for subtype-specific treatment [2], including *PDCD1LG2* locus for use of nivolumab and pembrolizumab in the Epstein-Barr virus-associated group, EGFR for cetuximab, panitumumab, nimotuzumab, or matuzumab treatment in the chromosome instability group and Aurora kinase A/B inhibitors for treatment of the genomically stable (GS) subgroup [3,4,5,6]. Immunohistochemical (IHC) has become a significant tool in clinical diagnostics and is frequently utilized to classify malignant cells [7]. In gastric cancer, a panel of six biomarkers was used in tumor stratification [8,9]. In a similar approach, cancers of the endometrium [10,11], lung [12], triple-negative breast [13], esophagogastric junction carcinomas [14] were stratified into discrete molecular classes using tumor-specific IHC-based biomarkers. Multiplexed IHC for the concurrent detection of a number of biomarkers in lung cancer is increasingly becoming point-of care in treatment [15]. Such translation of molecular information implies the feasibility of similar applications in other tumor types. High-grade serous ovarian cancer (HGSC) represents aggressive tumors characterized by swift metastatic progression and poor patient prognosis [16]. Despite radical surgery and initial response to platinum and taxane based chemotherapy, most patients relapse following median progression-free survival of ~18 months [17,18]. Clinical outcomes vary considerably emphasizing an imminent need to improve therapeutic options. Large-scale molecular analyses have recently identified diverse molecular pathways, mutations, gene expression, morphologies, cell(s) of origin, etc. leading to a systematic understanding of HGSC despite its heterogeneity [19,20,21,22,23]. Our earlier analyses of gene expression datasets also resolved three classes in HGSC that were associated with discrete mechanisms of metastases [24]. Development of targeted therapies now necessitates the establishment of a robust diagnostic pipeline for HGSC stratification. As a first step towards this aim, the present study evaluates the application of six markers using immunohistochemistry (IHC) and histochemistry (HC), the establishment of standard operating procedures (SOPs) and development of a reference human tissue library for these markers along with scoring guidelines for interpretation of marker expression. Further evaluation and application were performed in xenografts and commercial tissue microarrays (TMAs), along with the determination of thresholds for clinical classification in resected primary tumors and secondary metastases and/or cell blocks prepared from ascitic fluid of chemo-naïve and chemo-treated patients were also achieved (Supplementary Figure S1). These efforts define the establishment of diagnostic principles for application in clinical practice.

## 2. Materials and Methods

### 2.1. Sample Collection and Preparation

Formalin-fixed and paraffin-embedded (FFPE) tissue collection and processing using routine methods following surgery, after obtaining informed consent, were approved by the respective Institutional Review Board of NCCS with project identification code IEC/22/12/2014. All subjects gave their informed consent for inclusion before they participated in the study. The study was conducted in accordance with the Declaration of Helsinki, and the protocol was approved by the Ethics Committee of the National Centre for Cell Science IEC/22/12/2014. In all, retrospective 96 primary high-grade serous ovarian adenocarcinoma patient cases with information of name, age, grade, stage, and treatment status were selected, who had undergone surgery at the Armed Forces Medical College (Pune, India; 2008–2015), Tata Medical Centre (Kolkata, India; 2013–2014), Jehangir Hospital (Pune, India; 2003–2005), Command Hospital (Pune, India; 2010–2011) and Inlaks & Budhrani Hospital (Morbai Naraindas Budrani Cancer Institute, Pune, India; 2013–2015).

### 2.2. Animal Studies

Animal experimentation was in accordance with the rules and regulations of the National Centre for Cell Science (NCCS) Institutional Animal Ethics Committee. The study was approved with project number IAEC/2011/B-163. Xenografts were raised as described earlier [24]. In brief, 2.5 × 10^6^ cells of cell lines OVCAR3, OV90, OVMZ6, A4, CP70, PEO14, and CAOV3 were injected subcutaneously in non-obese diabetic/severe combined immunodeficient (NOD/SCID) mice. Animals were maintained under pathogen-free conditions and assessed every 2 days until the tumor diameter was ~1 cm, whereupon animals were sacrificed and tumors harvested.

### 2.3. Immunohistochemical (IHC) and Histochemical Staining (HC)

IHC and HC were performed in 5 μm sections of FFPE blocks fixed by drying at 60 °C for at least 1 h in an oven using standard protocol, deparaffinized in xylene and hydrated in ethanol-distilled water gradient. Heat-induced epitope retrieval (HIER) was carried out for 30 min at pH = 9/pH = 6 (Himedia, India). For peroxidase inactivation, sections were incubated in 3% H_2_O_2_ for 30 min (Qualigens, MA, USA), followed by 1× Blocking Solution for 10 min (Biogenex, CA, USA) and overnight incubation in primary antibody (Abcam, MA, USA; E-cadherin ready-to-use, Biogenex, CA, USA; Santa Cruz Biotechnology Inc.Texas, USA; Abcam, MA, USA). Sections were washed and incubated with anti-rabbit HRP-conjugate (Jackson ImmunoResearch Laboratories, Inc., PA, USA) or anti-mouse HRP-conjugate (Jackson ImmunoResearch Laboratories, Inc., PA, USA) for 1 h, and color developed with DAB (Thermo Pierce, MA, USA); hematoxylin used as a counterstain. These sections were dehydrated and mounted in DPX (Qualigens, MA, USA). Negative controls were prepared in the absence of primary antibody. IHC methods were standardized for each marker as Standard Operating Procedures (SOPs; Supplementary Dataset 1). SOPs were developed considering the positive and negative expression tissue controls, and secondary antibody control for each batch of IHC run. For HC-based HA detection, test sections were exposed to freshly prepared hyaluronidase (1 mg/mL; Sigma-Aldrich, MA, USA); control slides were incubated in phosphate buffer for 1 h at 37 °C. Sections were washed in running water for 10 min and stained with Alcian blue for 30 min (pH = 2.5, Fluka, MA, USA), counterstained with Nuclear Fast Red Solution for 2 min (Sigma-Aldrich, MA, United States), and dehydrated and mounted in DPX (Qualigens, MA, USA). Positive experimental controls included testis (TCF21, PARP1), liver (E-cadherin), lymphocytes (Slug), gall bladder (ANXA2), and small intestine (hyaluronan); negative controls included heart (TCF21, E-cadherin, Slug, ANXA2) and mucosa of the small intestine (PARP1). Slides for human tissues, xenografts, and TMA were reviewed independently; a consensus was reached to establish tissues for reference score.

### 2.4. Statistical Analysis

Each observer scored the biomarkers for frequency, intensity, and localization. Computation of these scores led to the derivation of biomarker and class indices, which compared between groups by Pearson correlation using SPSS (version 20, SPSS Inc., Chicago, IL, USA) for Windows to delineate classes. Student’s t-test and ANOVA were determined in Microsoft Excel 2016; *p* < 0.05 was considered significant.

## 3. Results

### 3.1. Selection of Class-Specific Biomarkers, Development of SOPs for Detection, a Reference Human Tissue Library and Guidelines for Scoring

**Figure 1:**
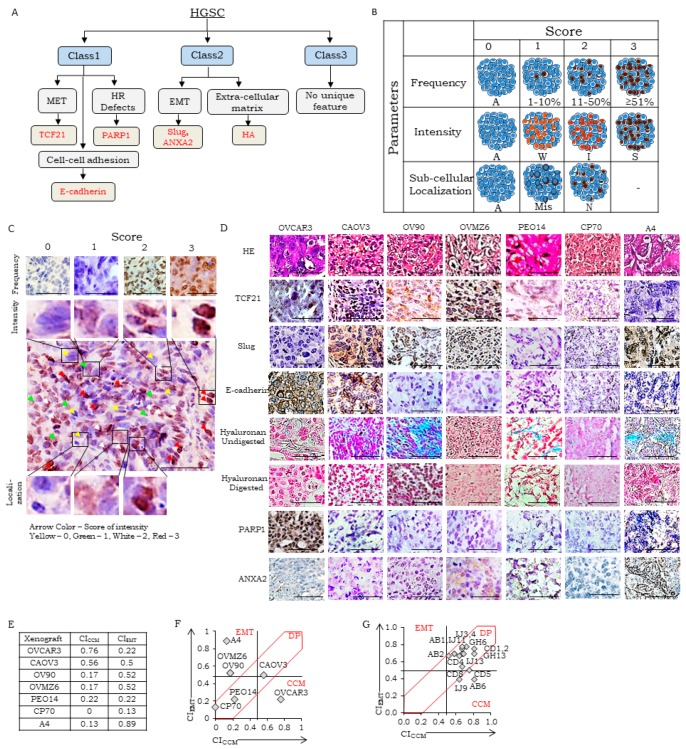
Class associations and marker scoring guidelines; (**A**) rationale for class-specific biological function based putative marker selection, MET: Mesenchymal-to-epithelial transition, HR: Homologous Recombination mediated DNA Damage Repair, EMT: Epithelial-to-mesenchymal transition; (**B**) schematic of scoring guidelines for IHC based staining of nuclear markers (TCF21, PARP1, Slug), A: Absent, W: Weak, I: Intermediate, S: Strong, Mis: Mislocalised, N: normal localization. A similar approach was used for scoring of membrane markers (E-cadherin, ANXA2) except that sub-cellular location was scored either 1 (cytoplasm) or 2 (cell membrane), while extracellular expression of hyaluronan fibers (evaluated as blue color developed by Alcian blue staining that is lost on hyaluronidase) was scored 1 in distant tumor stroma, and 2 in tumor epithelial cell nests. Scoring and analyses of marker expression in xenografts and TMAs; (**C**) Tissues and markers for Scoring of Frequency–0: A4 (TCF21), 1: OV90 (Slug), 2: OVCAR3 (PARP1), 3: A4 (Slug); scoring of intensity and localization-CAOV3 (TCF21), different regions representing scores of 0–3 (intensity) and 0–2 (localization), Scale bar is 50 µm; (**D**) Representative micrographs of HGSC xenografts for: Row 1-H&E (hematoxylin and eosin) stained section while Rows 2,3,4,7,8 represent IHC-based identification of TCF21, Slug, E-cadherin, PARP1, and ANXA2; Rows 5 and 6 represent HC-based identification of HA fibers in untreated and hyaluronidase digested xenograft sections respectively, scale bar is 50 µm; (**E**) class Indices of xenografts; (**F**,**G**) scatter plots of CI_CCM_ vs. CI_EMT_ in xenografts and high-grade serous ovarian carcinoma (HGSC) cases in TMA respectively. DP-double positive; CCM-cooperative cell migration.

A strong correlation of the transcription factors TCF21 and Slug with Class 1 (Cooperative Cell Migration/CCM-class) and Class 2 (Epithelial-to-Mesenchymal/EMT-class) tumors respectively [15] lent consideration to their inclusion in this study. E-cadherin was selected as a feature of cell-cell adhesion to substantiate CCM class-specific purported biological functions and PARP1 for defects in homologous recombination. Known associations of EMT with AnnexinA2 (ANXA2) and its interactions with Slug, and extra-cellular matrix components including hyaluronan (HA) and its synthesizing genes (*HAS1* and *HAS2*) suggested HA as a candidate marker (Supplementary Table S1, Supplementary Figure S2). Class 3 tumors lacked any unique biological features, hence no specific markers were assigned for their identification. An inability to correlate variations in Vimentin protein levels with either of the transcription factor present in these groups refrained its inclusion. The final screening biomarker panel thus comprised of TCF21, E-cadherin, PARP1, Slug, ANXA2, and HA (Figure 1A).

Evaluation of any novel marker in tumor stratification necessitates the establishment of standard operating protocols (SOPs) to address pre-analytic (slide coating, tissue selection, fixation, processing), analytic (clone and antibody selection, buffers and instruments for antigen retrieval, antibody/enzyme concentration, duration of incubations at each step, etc.), and post-analytic parameters (interpretation, analyses and reporting of expression in the reference and control tissues). These were established for our panel (Supplementary Dataset 1), along with the development of a reference library based on the Human Protein Atlas (HPA) [25] using appropriate normal human tissues. Three specific metrics associated with IHC/HC detection viz. frequency, intensity, and localization were applied in developing universal guidelines for marker scoring (Figure 1B depicts a schematic for transcription factor marker scoring, while a reference score sheet is provided in Supplementary Figure S3). The subjectivity of analyses due to inter-personal observation variation was minimized by collecting independent scores from five observers followed by a comprehensive pathology review to arrive at a consensus in case of difference in opinions. Specific scoring guidelines for each marker that were thereby agreed on and corresponding healthy tissue included the following:

(i) Score for Marker Frequency (S_Freq_)-percentage expression in total tumor cells of tissue section on a scale of 0–3 (0: absent, 1: 1–10%, 2: 11–50%, and 3: ≥51% marker-positive),
  - TCF21: cardiac myocytes, ovarian stromal cells, and germinal cells of testis represented S_Freq_ 0, 1, and 3 respectively; S_Freq_ = 2 could not be identified in healthy tissues.
  - E-cadherin: cardiac myocytes, liver hepatocytes, and prostate epithelial cells represented S_Freq_ 0, 2, and 3 respectively; healthy tissues representing S_Freq_ = 1 could not be identified.
  - PARP1: mucosa of the small intestine, cardiac myocytes, germinal basal cells of testis represented S_Freq_ as 0, 1, and 3 respectively; healthy tissues representing S_Freq_ = 2 could not be identified.
  - Slug: cardiac myocytes, smooth muscles of the appendix, lymphocytes of the small intestine represented S_Freq_ 0, 1, and 2 respectively; healthy tissues representing S_Freq_ = 3 could not be identified.
  - HA: cartilage and sub-mucosa of the small intestine represented S_Freq_ as 2 and 3 respectively; healthy tissues representing S_Freq_ = 0 or 1 could not be identified.
  - ANXA2: cardiac myocytes, the somatic muscle of the small intestine, epithelial cells of the gall bladder represented S_Freq_ 0, 1, and 3 respectively; healthy tissues representing S_Freq_ = 2 could not be identified.
(ii) Score for marker intensity (S_Int_)-intensity of brown stain for IHC and blue for HC in positively stained tissue sections. A scale of 0–3 was established, 0: absent, 1: weak, 2: moderate, and 3: strong intensity of marker-positive cells,
  - TCF21: cardiac myocytes, ovarian stromal cells, germinal basal cells of testis represented S_Int_ 0, 1, and 2 respectively; S_Int_ = 3 could not be identified in healthy tissues.
  - E-cadherin: cardiac myocytes, epithelial cells of the small intestine, epithelial cells of prostate represented S_Int_ 0, 2, and 3 respectively; healthy tissues representing S_Int_ = 1 could not be identified.
  - PARP1: mucosa of the small intestine, cardiac myocytes, and germinal basal cells of testis represented S_Int_ 0, 1, and 2 respectively; healthy tissues representing S_Freq_ = 3 could not be identified.
  - Slug: cardiac myocytes, smooth muscle of the appendix, and lymphocytes of the small intestine represented S_Int_ 0, 1, and 2 respectively; healthy tissues representing S_Int_ = 3 could not be identified.
  - HA: Intensity for hyaluronan was measured as blue color intensity developed by Alcian blue in comparison to hyaluronidase digested tissue section. Sub-mucosa of the small intestine and cartilage tissues represented S_Int_ 1 and 2 respectively; healthy tissues representing S_Int_ = 0 or 3 could not be identified.
  - ANXA2: cardiac myocytes and epithelial cells of gall bladder represented S_Int_ 0 and 2 respectively; healthy tissues representing S_Int_ = 1 or 3 could not be identified.
(iii) Score for Marker Localization (S_Loc_)-representing sub-cellular location of marker in the tissue section on a scale of 0–2, 0: Absent, 1: mislocalized (cellular localization does not correspond to known functionality, for example, cytoplasmic location for TCF21, PARP1, Slug, E-cadherin, ANXA2 or HA), 2: normal localization (for example, nuclear expression of TCF21, PARP1 or Slug, membrane for E-cadherin, membrane or cytoplasmic for ANXA2 and extracellular expression of HA.
  - TCF21: cardiac myocytes, liver hepatocytes, germinal basal cells of testis represented S_Loc_ 0, 1, and 2 respectively.
  - E-cadherin: cardiac myocytes, prostate epithelial cells represented S_Loc_ 0 and 2 respectively; healthy tissues representing S_Loc_ = 1 could not be identified.
  - PARP1: mucosa of the small intestine, germinal basal cells of testis represented S_Loc_ 0 and 2 respectively; healthy tissues representing S_Loc_ = 1 could not be identified.
  - Slug: cardiac myocytes, the somatic muscle of the appendix, lymphocytes of the small intestine, represented S_Loc_ 0, 1, and 2 respectively.
  - HA: cartilage represented S_Loc_ of score 2; healthy tissues representing S_Loc_ = 1 could not be identified. A further consensus was reached in the pathology review to consider extracellular staining in tumor nests that is eliminated following hyaluronidase treatment as a proper localization, while distant stroma-associated HA was considered as mislocalization.
  - ANXA2: cardiac myocytes, stromal cells of the gall bladder, epithelial cells of the gall bladder represented S_Loc_ as 0, 1, and 2 respectively.

### 3.2. Establishment of Scoring Guidelines for Stratification Using a Panel of Xenograft

Initial validation of the biomarker expression and scoring scheme was achieved using HGSC cell line derived xenografts generated in NOD/SCID mice (Figure 1C,D). TCF21 localization in xenograft sections was either dominantly nuclear (CAOV3 and PEO14), cytoplasmic (OVMZ6, OV90, and OVCAR3), or negligible (CP70 and A4); similarly, Slug was nuclear (OVMZ6, OV90, and A4), cytoplasmic (CAOV3 and CP70), or absent (OVCAR3 and PEO14). Moderate intensity of E-cadherin at the cell membrane was observed in ~50% tumor cells in CAOV3 and OVCAR3 xenografts but was lower in OVMZ6, CP70, OV90, A4, and PEO14 xenografts. Significantly, high-intensity expression of nuclear PARP1 was evident only in OVCAR3 xenografts; while other xenografts had significantly lower expression. High frequency, moderate intensity of HA was observed in CAOV3, OV90, and A4 xenografts, while OVCAR3 and PEO14 expressed HA at low to moderate frequency with weak intensity. Significant expression of ANXA2 was evident only in OVMZ6 and A4 xenografts. Consensus marker scores consolidated by the pathologist panel for each marker and xenograft (S_Freq_, S_Int_, S_Loc_; Table 1) were used to derive specific Biomarker Indices (BI; Equation (1)). Class-indices representing class-specific metrics of consolidated marker expression were derived from class-specific BI (CI_CCM_ and CI_EMT_; Equations (2) and (3) respectively; Table 1).

(1) $$BI=\frac{1}{3}\left(\frac{observed\;S_{Freq}}{max\;S_{Freq}}\right)+\frac{1}{3}\left(\frac{observed\;S_{Int}}{max\;S_{Int}}\right)+\frac{1}{3}\left(\frac{observed\;S_{Loc}}{max\;S_{Loc}}\right)$$

(2) $$CI_{CCM}=\frac{BI_{TCF21}+\;BI_{E-cadherin}+\;BI_{PARP1}}{\begin{array}{c} 3 \end{array}}$$

(3) $$CI_{EMT}=\frac{BI_{Slug}+\;BI_{HA}+\;BI_{ANXA2}}{\begin{array}{c} 3 \end{array}}$$

Class indices represent class-specific metrics of consolidated markers expression; CI_CCM_ and CI_EMT_ computed for xenografts ranged from (0–0.76) and (0.22–0.89) respectively (Table 1; Figure 1D). The distribution of median CI_EMT_ vs. CI_CCM_ (±10%) values were further applied in class identification. Thus, OVCAR3 represents CCM-class; A4, OVMZ6, and OV90 the EMT-class; CAOV3, PEO14, and CP70 being double positive (DP; Figure 1E). Such inclusiveness of expression of the six markers quantifies molecular heterogeneity in mixed/unclassified tumors and assigns biological functions to the ambiguous Class 3 through relative marker expression.

### 3.3. Evaluation of Stratification Guidelines in TMAs

The above biomarker scoring and class identification guidelines were applied to commercial TMAs (duplicate cores per sample) which included two normal ovary and 13 HGSC tumor cases among other ovarian cancer subtypes. Availability of limited consecutive slides (*n* = 5) led to screening of only four of the six biomarkers (TCF21, E-cadherin, Slug, and HA) and equations (ii) and (iii) were appropriately modified in consideration of a four marker-based class identification (Supplementary Figure S4; Supplementary Table S2). Biomarker score averages for clinical cases represented on TMAs were considered for computation of BI and CI values (of each core in duplicate showed a near-similar expression for all biomarkers). We observed that a majority of HGSC TMA-cores expressed high-intensity cytoplasmic TCF21, moderate nuclear expression of which was present in ~5–10% of tumor cells. Except in four cases, Slug expression was weak to moderate cytoplasmic and nuclear localization was evident in only 5–10% tumor cells. Moderate expression of E-cadherin at cell membranes was observed in nearly 50% of tumor cells, and extracellular HA fibers also stained at a moderate intensity in tumor cell nests. Consolidation of biomarker scores of each case, computation of BI and CI values followed by plotting the distribution of CI_CCM_ vs. CI_EMT_ values of TMA cores indicated three cases to represent CCM-Class while the remaining belonged to DP-Class; and EMT-Class remained unrepresented (Figure 1F).

### 3.4. Evaluation of Clinical Samples Associates CCM-Markers with Metastases and Chemotherapy

The variance in frequencies of class profiles between xenografts and TMAs emphasized the pertinence of screening larger numbers of clinical samples in validation. Towards assessing clinical representation, we obtained and stratified 160 tumor samples pathologically diagnosed as HGSC from 96 patients. These included primary (ovary (T), fallopian tube (FT)) and metastatic tumors (omentum (O), peritoneal ascites-derived cell blocks (A); Supplementary Table S3). BI and CI scores with CI_CCM_ and CI_EMT_ values for each tumor were computed from marker scores (Supplementary Tables S4–S8) followed by evaluation of CI distribution towards class assignment as performed for xenografts and TMA. Data analysis of this clinical cohort was conducted in tumor groups as given Table 2.

**Figure 2:**
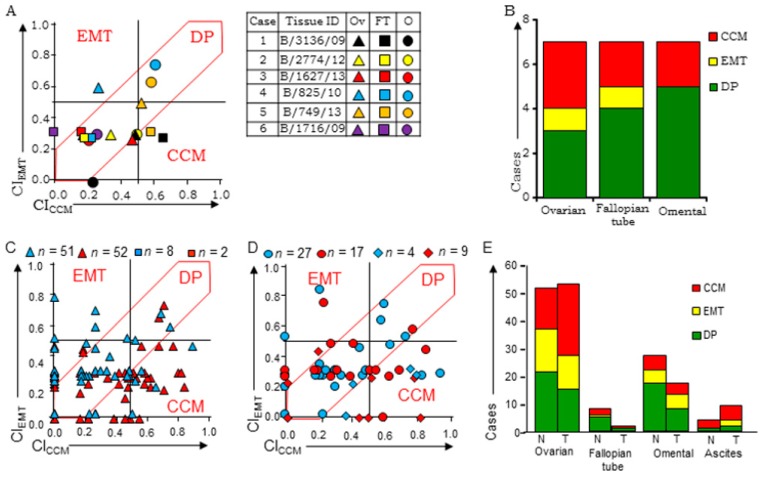
(**A**) Scatter plot of CI_CCM_ vs. CI_EMT_ distribution for chemo-naïve cases with tumors detected in ovary (Ov), fallopian tube (FT), and omentum (O) (left panel), and a reference case-chart (right panel), (**B**) graphical representation of class-specification of Group A tumors, (**C**) scatter plots of CI_CCM_ vs. CI_EMT_ distribution for single chemo-naïve or -treated (red and blue shapes respectively) tumors from-ovary △ & fallopian tube ⬜, (**D**) omentum ⭘ and ascites cell blocks ◇, (**E**) graphical representation of Group B tumors (chemo-naïve-N; chemo-treated-T). EMT-epithelial to mesenchymal transition; DP-double positive; CCM-cooperative cell migration.

Examination of ‘Group A’ tumors representing different sites of metastases and stages of tumor progression in six chemo-naïve cases revealed consistent class-associations across different sites in two cases (B/2774/12 and B/3136/09), while suggesting class switching in the remaining four cases wherein marker expression was altered in following metastases (Figure 2A). One of the latter four cases expressed CCM-markers in ovarian tumors; FT and omental tumors were DP-Class (B/1627/13). The ovarian tumor of case B/825/10 expressed EMT-markers that switched to DP-Class in FT and omental tumors. Ovarian and omental tumors of the remaining two cases segregated into DP-Class, while FT tumors expressed either CCM (B/749/13) or EMT (B/1716/09) markers. Overall, the three tumor sites predominantly segregated into CCM or DP Class; only one case of ovarian and FT tumors was represented as EMT-Class (Figure 2B; Supplementary Tables S9 and S10).

CI-based class-assignment in ‘Group B’ tumors stratified chemo-naïve ovarian tumors into DP, CCM or EMT classes (33.3%, 29.4%, and 29.4% respectively; Figure 2C,E), while treated ovarian tumors exhibited dominant representation of CCM-Class with lower frequencies of EMT and DP classes (48.0%, 23.0%, and 19.2% respectively). Chemo-naïve FT tumors stratified into CCM, EMT or DP Class (*n* = 2, 1, and 8 respectively; Figure 2C,E), while those after treatment belonged to either CCM or DP Class. Omental tumor deposits were predominantly DP-Class, with a marginal increase in the frequency of CCM and EMT markers in treated samples (Figure 2D,E), while chemo-naïve as well as treated ascites cell blocks presented more frequently with the CCM-class (Figure 2D,E). Overall, ‘Group B’ patient tumors show predominantly CCM or DP expression (Supplementary Table S11) as compared with either xenografts (mostly EMT-class) or TMAs (CCM and DP class). These findings support metastasis/chemotherapy-induced HGSC tumor expression towards a CCM subtype.

### 3.5. HGSC Tumors at Different Sites Exhibit Molecular Heterogeneity and Class-Switching

**Figure 3:**
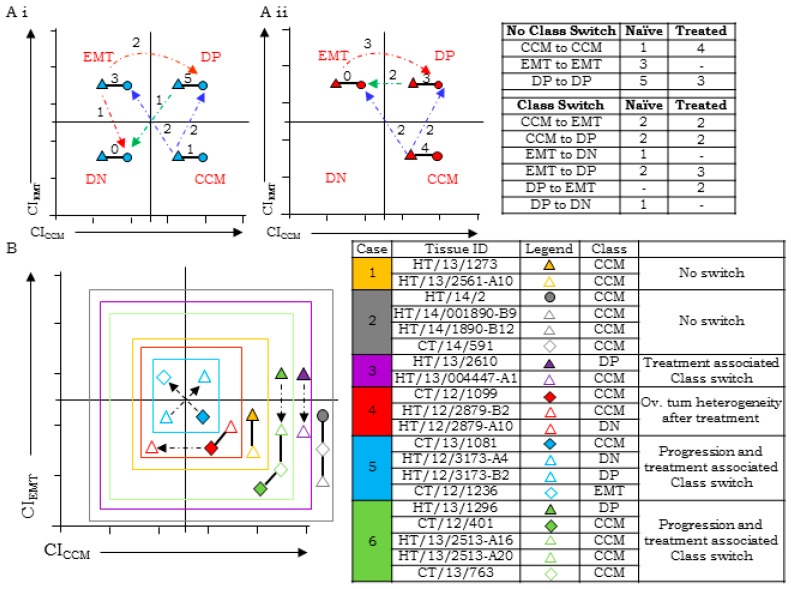
Class switching detected in cases with (**A**) ovarian and omental tumors tissues of (i) chemo-naïve (*n* = 17) and (ii) chemo-treated (*n* = 16) patient cohort; (**B**) class switching in tumors collected from patients (*n* = 6) prior to chemo-treatment (filled shapes) and post chemo-therapy (empty shapes) as determined through ascites (diamond), primary tumor (triangle), and omentum (circle). EMT-epithelial to mesenchymal transition; DP-double positive; DN-double negative; CCM-cooperative cell migration.

Class switching during tumor progression from ovarian to omental sites was further examined in ‘Group C’ samples that comprised of tumors from either chemo-naïve (*n* = 17) or -treated cases (*n =* 16). Almost half of the cases in both groups did indeed exhibit metastases associated class-switching (Figure 3(Ai,Aii); Supplementary Figure S5A), although lack of a specific direction to the switch possibly suggests the involvement of other factors in the determining marker expression.

The last analytical set of Group D tumors comprised of six ovarian, ascites, and/or omental tumor pairs before and after therapy (Figure 3B; Supplementary Figure S5B). Considering the limited cases that represent unique behavior, we have discussed them on a case to case basis. Cases 1 and 2 strongly conformed to CCM-class after disease progression as well as treatment, while the remaining four cases exhibited class-switching. Chemotherapy in Case 3 resulted in enhanced expression of CCM-markers over a DP-profile in untreated ovarian tumors, while Case 4 was associated with heterogeneity of marker expression following treatment. Case 5 was the most complex of all six and showed considerable marker heterogeneity between different tumor sites. Case 6 exhibited progression and therapy-associated class-shift towards the CCM class from a DP ovarian tumor. These findings further support the class-switching of HGSC tumors upon metastases and/or chemotherapy.

### 3.6. Disease Progression is Inclined Towards Enrichment of CCM-Markers

**Figure 4:**
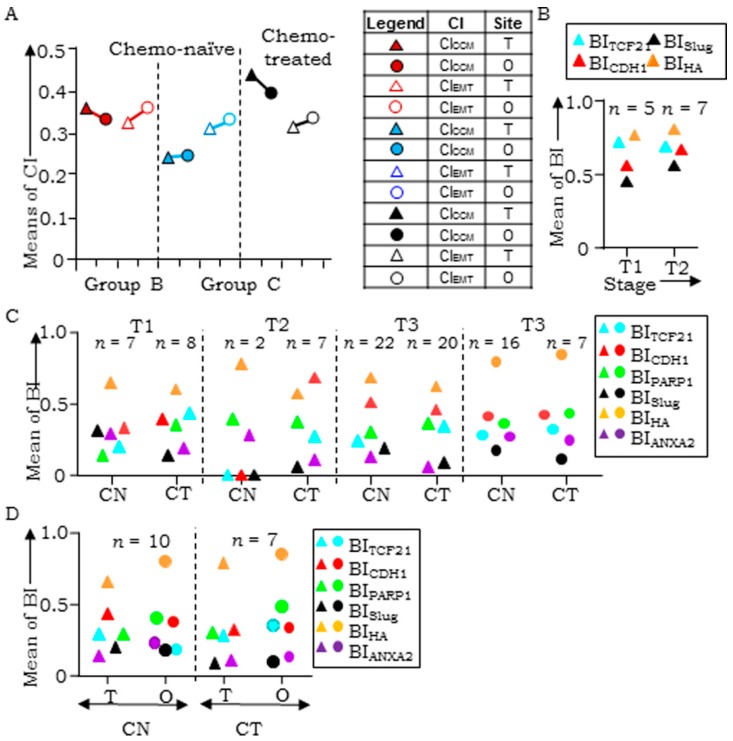
HGSC progression-associated marker expression. (**A**) Plot comparing CI_CCM_ with CI_EMT_ of tumors of Groups B and C respectively; (**B**) plot comparing biomarker (BI) scores for TCF21, E-cadherin, Slug, and HA in chemo-naïve ovarian tumors present in TMA for stages T1 and T2; (**C**) plot for chemo-naïve (left) and treated (right) paired ovarian (T)-omental (O) tumors; (**D**) plot for chemo-naïve (CN) and treated (CT) tumors at stages T1, T2, and T3 in ovarian △ and omental ⭘ tumors.

To evaluate the effects of therapy and disease progression vis-à-vis metastases and stage advancement on stratification, we further compared the means of CI_CCM_ and CI_EMT_ (M-CI_CCM_ and M-CI_EMT_ respectively) of ovarian, FT, and omental tumors from the same patient in chemo-naïve (CN) vs. chemo-treated (CT) groups. M-CI_CCM_ of CN ovarian-omental (T-O) tumors (Group ‘C’) was lower than that of CN ovarian-FT-omental (T-F-O) tumors (Group ‘A’), while M-CI_CCM_ of CT T-O tumors was enhanced (Figure 4A, Supplementary Table S12). Interestingly, M-CI_EMT_ of ovarian as well as omental tumors were similar between CN T-O vs. T-F-O tumors, and between CN T-O vs. CT T-O groups suggesting minimal effects of either disease progression or therapy on expression of EMT-markers. Towards elucidation of individual biomarker contribution likely to contribute to these differences, we analyzed their expression through means of BI (M-BI) during stage progression in CN HGSC cases represented on TMAs (T1 to T2) and in patient tumors. Nearly steady M-BI of TCF21, increased Slug, E-cadherin, and HA levels were revealed in the TMAs, with Slug expression being maximal at the T2 stage (*p* < 0.007; Figure 4B). Similar analyses of Group ‘B’ cases with available tumor stage information in CN (31-T, 1-FT, 17-O) and CT cases (35-T, 7-FT, and 9-O) were performed. CN T samples expressed comparable M-BI levels of TCF21 and HA, higher E-cadherin and PARP1 and lower Slug and ANXA2 at T3 over T1 stage; CT T samples had almost comparable M-BI profiles as CN tumors (Figure 4C, Supplementary Figure S6). Limited FT and O tumors at stages T1 and T2 restricted their analyses; CN- and CT-O tumors at stage T3 expressed comparable levels of markers. Group C CN O samples at stage T3 were associated with decreased TCF21 and E-cadherin concurrent with increased PARP1, ANXA2, and HA M-BI profiles (Figure 4D). CT T tumors had low M-BI scores for TCF21, PARP1, and HA in comparison to O and similar levels of E-cadherin, Slug, and ANXA2. CT T samples in this group displayed steady levels of TCF21 and PARP1, lower E-cadherin, Slug, and ANXA2 and increased HA levels over CN tumors; while treatment was associated with higher BI levels of TCF21, PARP1, and HA and lower levels of E-cadherin, Slug, and ANXA2 in the O tumors. This suggests that selected biomarkers play a significant role during disease progression.

**Figure 5:**
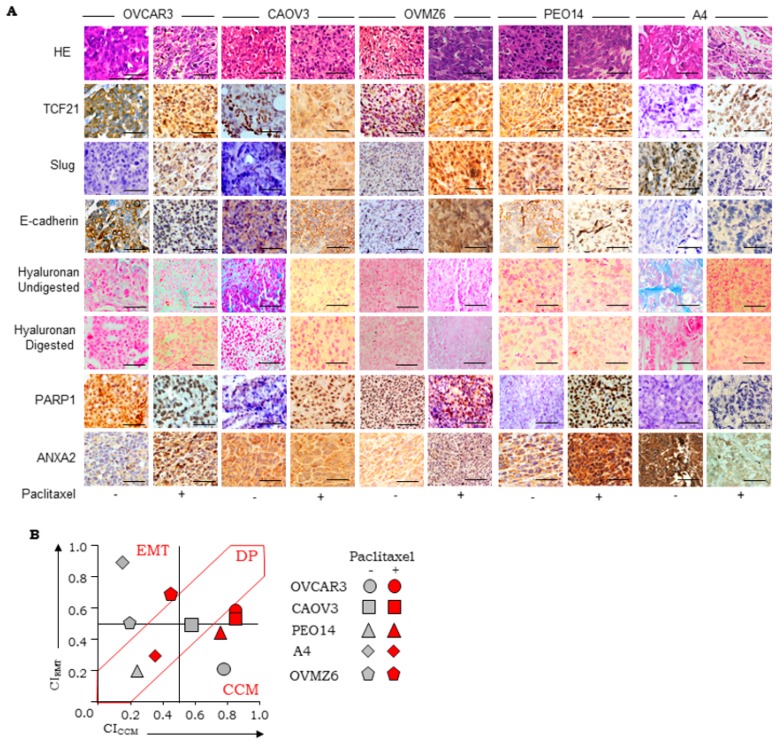
(**A**). Paclitaxel exposure alters scoring marker panel in HGSC cell line derived xenografts. Representative images of HGSC xenograft (control and paclitaxel treated) sections for Row 1-HE (hematoxylin and eosin), Rows 2,3,4,7,8 represent IHC-based detection of TCF21, Slug, E cadherin, PARP1, and ANXA2, Rows 5 and 6 represent HC-based identification of HA fibers in untreated and hyaluronidase digested sections respectively, scale bars-50 μm; (**B**) scatter plots of CI_CCM_ vs. CI_EMT_ derived from xenograft (control-grey and paclitaxel treated-red) scoring. EMT-epithelial to mesenchymal transition; DP-double positive; CCM-cooperative cell migration.

Class-switching in paired samples led us to examine similar effects of a chemotherapeutic challenge (paclitaxel) in the three classes representing cell lines derived xenografts viz. CCM-Class (OVCAR3), EMT-Class (A4, OVMZ6), and DP-Class (CAOV3, PEO14). Distribution of CI_CCM_ and CI_EMT_ values revealed a DP to CCM (CAOV3, PEO14) and EMT to DP (A4, OVMZ6) class switch. An outlier was the OVCAR3 xenograft (CCM class) that despite an increased CI score, provided no evidence of class switching (Figure 5A,B).

### 3.7. Correlation Between Transcript- and Protein-Based Stratification

**Figure 6:**
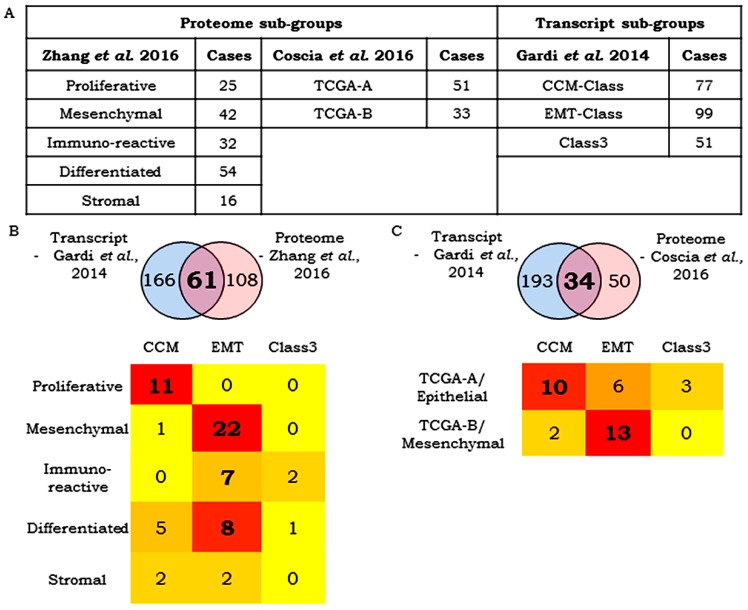
Comparison of HGSC stratification approaches. (**A**) Proteomics-based TCGA HGSC tumors stratification in 5 sub-groups by Zhang et al. 2016 (169 cases) and Coscia et al. 2016 (84 cases) and transcriptomics based HGSC stratification into three classes (Gardi et al. 2014); (**B**) comparison of Gardi et al. 2014 vs. Zhang et al., 2016; (**C**) comparison of Gardi et al. 2014 vs. Coscia et al. 2016.

Proteome-based profiling of TCGA samples has been reported earlier (Figure 6A) [26,27]. Comparison of tumor samples common to our earlier study and the five proteomic subtypes (*n* = 61; Refs. 24 vs. 26) indicated correlation between CCM and proliferative groups, while EMT tumors were dominantly mesenchymal, with a few being either immunoreactive or differentiated (Figure 6B); surprisingly, the stromal class had negligible associations. A similar comparison of tumor samples common to the transcript study and two proteomic subtypes (*n* = 34) [24,27] correlated the CCM-Class with TCGA-A/Epithelial cluster and EMT-Class with TCGA-B/mesenchymal cluster (Figure 6C). These observations indicate some degree of variation that could arise from differences between transcriptomic and protein abundances.

## 4. Discussion

Molecular histology is a convenient tool in biomarker discovery, evaluation, and validation that could facilitate personalized therapeutic choices [28,29]. In the present study, we focused on evaluating our previous molecular stratification [24] through the establishment of reproducible SOPs and scoring guidelines for six biomarkers in xenografts (TCF21, E-cadherin, PARP1, Slug, ANXA2, and HA; Phase 0), and partial validation in TMAs (Phase I) and clinical samples (Phase II). In a routine pathology analysis, incorrect biomarker sub-cellular localization is usually ignored or considered an artifact. This is in contrast to several cells and macromolecular studies that attribute altered cellular functions to mislocalized proteins especially in the context of transformation that suggests different biological functions [30,31]. The inclusion of this parameter for biomarker evaluation was hence considered essential in the present study along with frequency and intensity. Results were interpreted based on individual scores and by deriving a relation between them. Individual biomarker scores were consolidated to derive CCM and EMT class specific indices that were applied for tumor stratification. The dominance of EMT-class in cell line-derived xenografts, DP-class in TMAs representing human cases and CCM-class in clinical tumor samples were observed. These differences might reflect on the purported cell culture driven mesenchymal phenotype [32,33] and expression of EMT markers in xenografts. The results suggest cross-talk between transcription factors TCF21 with Slug in regulating intrinsic cellular states and tumor subtypes. Importantly, we achieved tumor stratification through the incorporation of features of intermediate phenotypes that effectively accounts for tumor heterogeneity. Phenotypic transitions captured through different cell lines representing different phenotypes is a significant step towards understanding tumor heterogeneity. The existence of cellular plasticity has been attributed in cancers of the lung [34], ovary [35], pancreas [36], and prostate [37], that substantiates the presence of phenotypic heterogeneity in tumors. Likewise, restricted tissue sampling and representation of heterogeneity in TMAs could lead to incomplete tumor evaluation [38]. Therapy-influenced heterogeneity of molecular expression reported earlier in multi-drug resistant cancers [39,40,41,42] was noted as occasional class switching in the present study wherein tumors in the same patient stratified into different classes either during disease progression or following chemotherapy. We believe that these effects reflect the influences of a new/altered niche on molecular expression in the same tumor [43,44]. Analyses of a larger patient cohort could clarify the interplay of protein expression during treatment procedures.

Any improvement in the accuracy of current triaging using new biomarkers thereby is a likely value-addition in optimizing the selection of the right therapeutic drugs and regimens in patients. Our findings now set the stage for evaluation of class-specific inhibitors in this direction. Olaparib, Niraparib, Veliparib, and Rucaparib that are in different phases of research and clinical trials for cancers including HGSC may be evaluated in the CCM-Class for PARP1 as a potential therapeutic target [45,46] (ClinicalTrials.gov Identifier: NCT00535119, NCT00664781, and NCT00516373). In contrast, the PI3K-Akt signaling pathway driven EMT in ovarian cancer [47] suggests evaluation of PI3K inhibitors like BKM120 or BYL719 in patients presenting with this class of tumors; Phase I clinical trials for both these molecules is underway for recurrent TNBC and HGSC [ClinicalTrials.gov Identifier: NCT01623349]. While tumors segregating in Class 3 need further research for molecular target identification, the DP-class may be evaluated for efficacy of either PARP1 or PI3K inhibitors or a combination of both [48,49,50,51]. Thus, we hope that CCM-Class and EMT-Class tumors would respond specifically upon treatment with PARP1 inhibitors and PI3K-Akt inhibitors, respectively. However, this has to be substantiated by specific clinical studies. In conclusion, the current study establishes the diagnostic principles and possibilities for molecular stratification in HGSC to address a few missing steps in achieving a bench to bedside translation.

## Figures and Tables

**Table 1:** Scores, biomarker and class indices (BI and CI respectively) for cooperative cell migration (CCM) and epithelial-to-mesenchymal transition (EMT) markers in xenografts.

**CCM Markers**													
**Cell Line Derived Xenograft**	**TCF21**				**E-cadherin**				**PARP1**				**CI_CCM_**
	**S_Freq_**	**S_Int_**	**S_Loc_**	**BI_TCF21_**	**S_Freq_**	**S_Int_**	**S_Loc_**	**BI_CDH1_**	**S_Freq_**	**S_Int_**	**S_Loc_**	**BI_PARP1_**	
CAOV3	2	2	2	0.78	3	2	2	0.89	0	0	0	0	0.56
OVMZ6	2	1	1	0.5	0	0	0	0	0	0	0	0	0.17
CP70	0	0	0	0	0	0	0	0	0	0	0	0	0
OV90	2	1	1	0.5	1	2	1	0.5	0	0	0	0	0.17
A4	1	1	1	0.39	0	0	0	0	0	0	0	0	0.13
OVCAR3	2	3	1	0.72	2	2	2	0.78	2	2	2	0.78	0.76
PEO14	1	2	2	0.67	0	0	0	0	0	0	0	0	0.22
**EMT Markers**													
**Cell Line Derived Xenograft**	**Slug**				**HA**				**ANXA2**				**CI_EMT_**
	**S_Freq_**	**S_Int_**	**S_Loc_**	**BI_Slug_**	**S_Freq_**	**S_Int_**	**S_Loc_**	**BI_HA_**	**S_Freq_**	**S_Int_**	**S_Loc_**	**BI_AnxA2_**	
CAOV3	3	1	1	0.61	3	2	2	0.89	0	0	0	0	0.5
OVMZ6	2	1	2	0.67	0	0	0	0	3	2	2	0.89	0.52
CP70	1	1	1	0.39	0	0	0	0	0	0	0	0	0.13
OV90	1	1	2	0.56	3	3	2	1	0	0	0	0	0.52
A4	3	3	2	1	3	2	2	0.89	3	1	2	0.78	0.89
OVCAR3	0	0	0	0	2	1	2	0.67	0	0	0	0	0.22
PEO14	0	0	0	0	2	1	2	0.67	0	0	0	0	0.22

**Table 2:** Distribution of clinical cohort in tumor groups.

Group	Analyses	Samples (n)
A	Between-group analyses of tumors in chemo-naïve tumors (T vs. FT vs. O)	6
B	Within the group of single tumors derived from either ovarian or FT sites, omental deposits or cell blocks from tumor ascites in chemo-naïve (CN) cases and chemo-treated (CT) cases	CN–51 (T), 8 (FT), 27 (O), 4 (A); CT–52 (T), 2 (FT), 17 (O), 2 (A)
C	Within groups of primary tumor & omental tumors pairs from either chemo-naïve (CN) or chemo-treated (CT) cases	CN–17; CT–16
D	Between-group analyses of tumor samples of the same case before and after chemotherapy	6

## Supplementary Materials

The following are available online at https://www.mdpi.com/2077-0383/8/3/330/s1, Figure S1: Flowchart of the approach for molecular stratification of HGSC tumors, Table S1: Class-specific enrichment of extracellular matrix (ECM)-associated genes, Figure S2: Heatmap representing class distribution of ECM-genes in the 3 Classes of TCGA-HGSC samples, Dataset 1: Standard Operating Procedures for IHC Detection of TCF21, E-cadherin, PARP1, Slug, ANXA2, and Histochemical detection of Hyaluronic Acid, Figure S3: Reference tissue expression controls of scoring guidelines, Figure S4: Representative TMA case for CCM-Class and DP-Class, Table S2: Biomarker and Class Indices for normal and HGSC cases in TMA leads to Class identification, Table S3: Distribution of tumor tissues obtained from different sites in 96 clinical HGSC cases, Table S4: CI scores for chemo-naïve cases in unpaired ovarian tumors and omental tumors leading to Class assignment, Table S5: CI scores for chemo-naïve cases in ovarian tumors paired with omental tumor and fallopian tumor, and tumor collected with ascites leading to class assignment, Table S6: CI scores for chemo-treated cases in unpaired ovarian tumors or omental tumor or ascites cell block leading to Class assignment, Table S7: CI scores for chemo-treated cases in ovarian tumors paired with omental tumor or ascites, fallopian tumor and FT with ascites leading to Class assignment, Table S8: CI scores for chemo-naïve and chemo-treated pair cases, Table S9: Class comparison of tumors of ovary, fallopian tube, omentum, and ascites cell block, Table S10: CI scores for chemo-naïve ovarian tumors paired with tumors of fallopian tube and omentum, Table S11: Class comparison of tumors of ovary, fallopian tube, and omentum, Figure S5: Scatter plot for tumors of chemo-naïve and treated ovary, fallopian tube, and omentum and pre-post pairs, Table S12: Group analysis of tumors of Group ‘B’ and ‘C’ ovary, fallopian tube and omentum stratified into respective class for chemo-naïve and chemo-treated cases, Figure S6: Effect of marker expression with Stage of HGSC at presentation.
