# Supplementary material for: Clinical Stratification of High-Grade Ovarian Serous Carcinoma Using a Panel of Six Biomarkers
Source: J Clin Med. 2019 Mar 8;8(3):330. doi: 10.3390/jcm8030330 (PMC6463261; doi:10.3390/jcm8030330)
Supplement: Supplementary file 1 [file jcm-08-00330-s001.pdf]

Supplementary Data

No.	Description	Page No.
1.	Supplementary Figure S1. Flowchart of approach for molecular stratification of HGSC tumors	1
2.	Supplementary Table S1. Class-specific enrichment of extracellular matrix (ECM)-associated genes	2
3.	Supplementary Figure S2.Heatmap representing class distribution of ECM-genes in the 3 Classes of TCGA-HGSC samples	3
4.	Supplementary Dataset 1. Standard Operating Procedures for IHC Detection of TCF21, E-cadherin, PARP1, Slug, ANXA2 and Histochemical detection of Hyaluronic Acid	4-27
5.	Supplementary Figure S3. Reference tissue expression controls of scoring guidelines	29
6.	Supplementary Figure S4. Representative TMA case for CCM-Class and DP-Class	30
7.	Supplementary Table S2. Biomarker and Class Indices for normal and HGSC cases in TMA leads to Class identification	31
8.	Supplementary Table S3. Distribution of tumor tissues obtained from different sites in 96 clinical HGSC cases	31
9.	Supplementary Table S4. CI scores for chemo-naïve cases in ovarian tumors paired with omental tumor and fallopian tumor, and tumor collected with ascites leading to class assignment	32
10.	Supplementary Table S5. CI scores for chemo-naïve cases in unpaired ovarian tumors and omental tumors leading to Class assignment	33
11.	Supplementary Table S6. CI scores for chemo-treated cases in unpaired ovarian tumors or omental tumor or ascites cell block leading to Class assignment	33
12.	Supplementary Table S7. CI scores for chemo-treated cases in ovarian tumors paired with omental tumor or ascites, fallopian tumor and FT with ascites leading to Class assignment	33-34
13.	Supplementary Table S8. CI scores for chemo-naïve and chemo-treated pair cases	35
14.	Supplementary Table S9. Class comparison of tumors of ovary, fallopian tube, omentum and ascites cell block	35
15.	Supplementary Table S10. CI scores for chemo-naïve ovarian tumors paired with tumors of fallopian tube and omentum	35
16.	Supplementary Table S11. Class comparison of tumors of ovary, fallopian tube and omentum	36
17.	Supplementary Figure S5. Scatter plot for tumors of chemo-naïve and –treated ovary, fallopian tube and omentum and pre-post pairs	36
18.	Supplementary Table S12. Group analysis of tumors of Group 'B' and 'C'	37

	ovary, fallopian tube and omentum stratified into respective class for chemo-naïve and chemo-treated cases	
19.	Supplementary Figure S6. Effect of marker expression with Stage of HGSC at presentation	38
20.	Additional references	39

Supplementary Figure S1. Flowchart of approach for molecular stratification of HGSC tumors

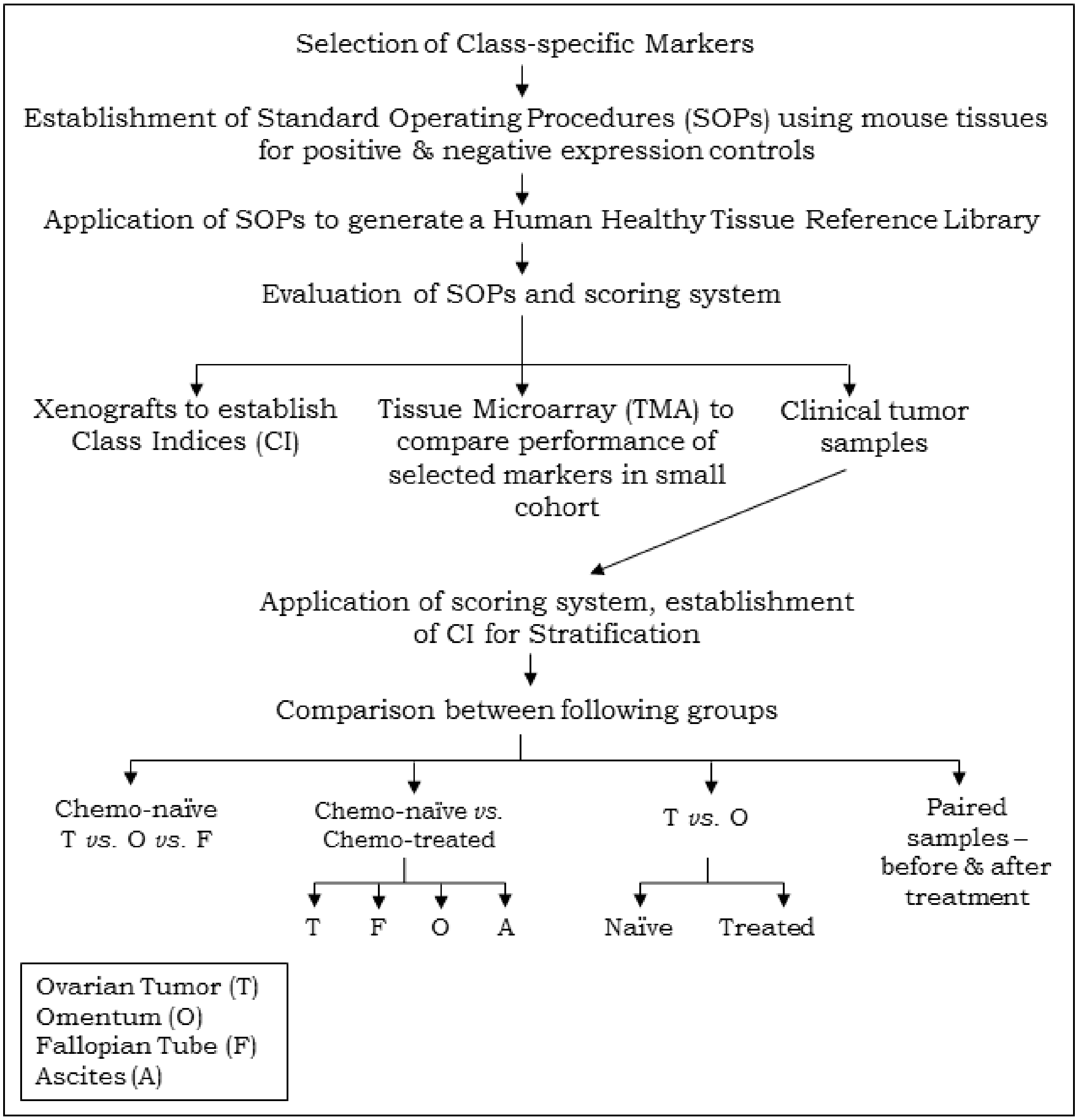

Supplementary Table S1. Class specific enrichment of extracellular matrix (ECM) – associated genes

Function	Gene			
**Proteins**	PAPLN	DAG1	FRAS1	USH2A
**Glycoproteins**	MUC1	TNN	LAMC1	MUC13
	MUC4	TNR	LAMC2	LAMA3
	MUC5B	FN1	LAMC3	SPARC
	MUC6	LAMA1	NID1	LAMB3
	MUC7	LAMA2	NID2	TNC
	NTN1	LAMA4	LAMA5	LAMB1
	NTN4	THBS1	THBS2	THBS3
	NPNT	MUC15	MUC16	MUC17
	MUC20	HYAL1	LAMB2	THBS4
**Proteoglycan**	SDC1	ASPN	HSPG2	GPC3
	SDC2	ECM2	AGRN	GPC4
	SDC3	FMOD	SPOCK1	GPC5
	SDC4	LUM	SPOCK2	GPC6
	TGFBR3	PREPL	SPOCK3	BCAN
	CD44	KERA	SPARC	DCN
	CSPG5	OMD	PTPRZ1	BGN
	ACAN	BCAN	NCAN	COL9A2
	GPC1	EPYC	COL9A3	COL18A1
	VCAN	OPTC	OGN	CHAD
	NYX	TSKU	PODN	PODNL1
	COL9A1	COL15A1		
**Non-Proteoglycan poly-saccharides**	HAS1	HAS2	HAS3	ACAN
	TNFAIP6	HMMR	NCAN	CD44
	VCAN			
**Fibrous protein**	COL1A1	COL4A3	COL17A1	COL5A3
	COL2A1	COL4A4	COL9A1	COL9A2
	COL3A1	COL4A5	COL9A2	COL9A1
	COL5A1	COL4A6	COL14A1	COL13A1
	COL5A2	COL8A1	COL19A1	COL17A1
	ELN	POSTN	COL6A3	COL6A6
	COL15A1	COL18A1	COL4A1	COL4A2
	FBN1	FBN2	FBLN1	FBLN2
	COL8A2	COL10A1	COL8A1	COL8A2
	COL6A1	EMILIN1	COL6A2	FBLN5

Class1	Class2		Class 3
COL18A1	ACAN	NTN4	FMOD
COL2A1	ASPN	OGN	PTPRZ1
COL4A6	BGN	OMD	HYAL1
COL9A3	CD44	PODN	SDC3
FRAS1	COL10A1	PODNL1	LAMB2
GPC1	COL15A1	POSTN	
GPC3	COL1A1	SDC1	
LAMA1	COL3A1	SDC2	
LAMA2	COL4A1	SDC4	
THBS4	COL4A2	SPARC	
	COL5A1	SPOCK1	
	COL5A2	THBS1	
	COL5A3	THBS2	
	COL6A1	TNC	
	COL6A2	TNFAIP6	
	COL6A3	TNN	
	COL6A6	TSKU	
	COL8A1	VCAN	
	COL8A2		
	DCN		
	ECM2		
	ELN		
	EPYC		
	FBLN2		
	FBLN5		
	FBN1		
	FN1		
	GPC6		
	HAS1		
	HAS2		
	LAMA4		
	LAMB1		
	LUM		
	MUC1		
	NID2		

Supplementary Figure S2. Heatmap representing class distribution of ECM-genes in the 3 Classes of TCGA-HGSC samples

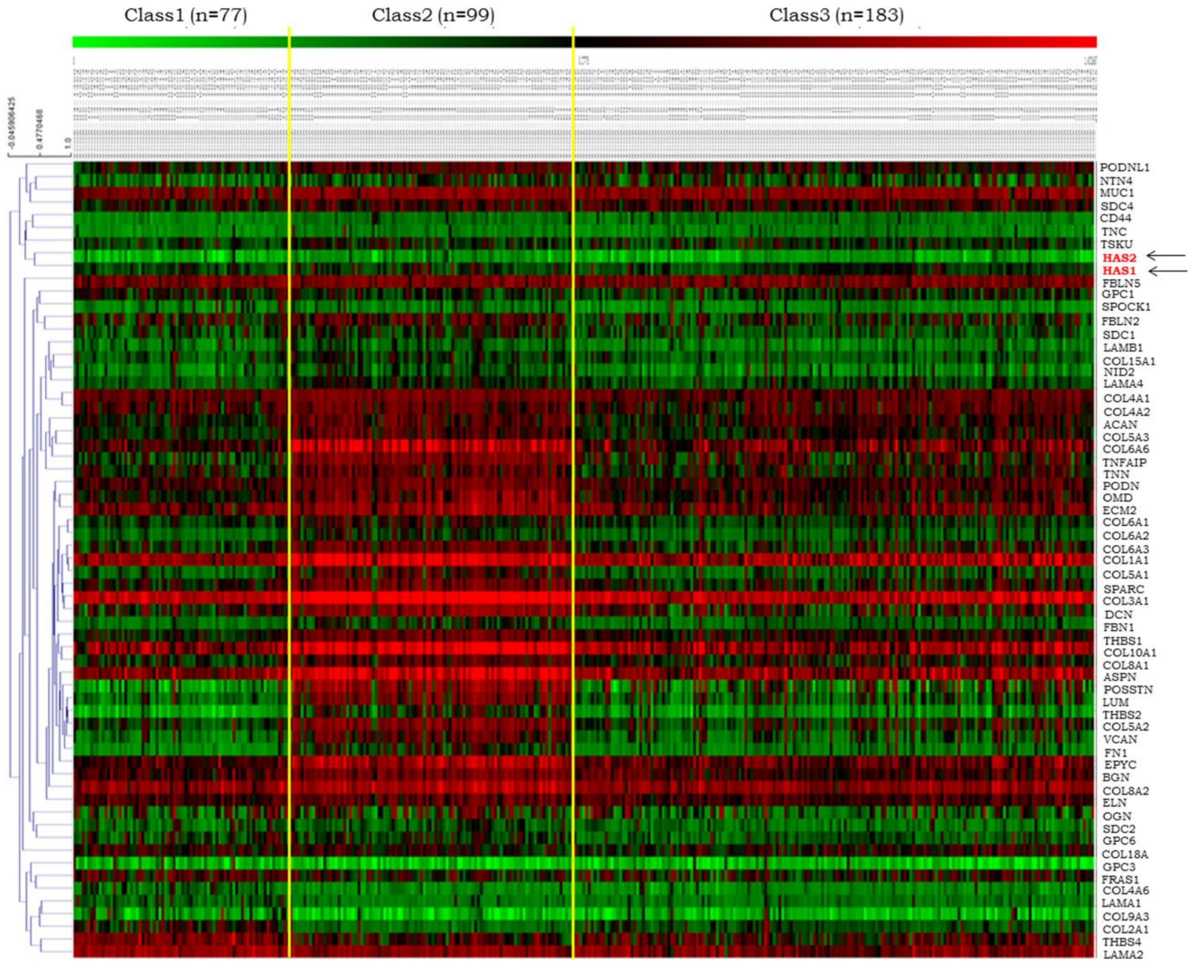

Supplementary Figure S2. Heatmap representing class distribution of ECM-genes in the 3 Classes of TCGA-HGSC samples. Class 1 (n=77), Class 2 (n=99), Class 3 (n=183).

Supplementary Dataset 1. Standard Operating Procedures for IHC Detection of TCF21, E-cadherin, PARP1, Slug, ANXA2 and Histochemical detection of Hyaluronic Acid

To increase the robustness of IHC-HC analyses, we developed SOPs for individual markers. Pre-analytic phase parameters included immediate tissue fixing in formaldehyde-based fixative and use of fresh solutions for tissue processing (normal mouse and human tissues and xenografts). Double coating of slides with poly-L-lysine solution improved adherence of tissues, additional heating at 60ºC for 1h curtailed tissue dislodgement during subsequent processing. Other factors including instrumentation used for tissue processing and block preparation; number of post-fixation washes and duration during tissue processing; and block storage conditions and durations did not influence IHC outcomes as long as tissue did not dry during processing.

Analytic phase features including clone of antibody, buffers involved in antigen retrieval (AR), antibody concentrations and incubation times were governed by protein under study. Polyclonal antibodies against TCF21 and Slug (#ab32981 and #ab27568 respectively, Abcam, Cambridge, MA, USA) and in-house monoclonal antibody mAb150 developed against ANXA2 worked ideally along with citrate buffer (pH 6.1), while E-cadherin and PARP1 immunostaining was optimum with Tris-EDTA buffer (pH 9.0) following Heat-induced Epitope Retrieval (HIER). Further, HIER performed in automated pressure cooker designed for IHC was more effective as compared with microwave based AR in view of avoiding excess evaporation of HIER buffer and tissue drying. A comparison between concentrated and pre-diluted E-cadherin antibodies (#HPA004812, Sigma-Aldrich, Billerica, Massachusetts, USA and #AM390-5M, Biogenex, Fremont, CA, USA respectively) showed better staining with pre-diluted antibody as compared with concentrated antibody and hence was selected for subsequent analytical staining. The optimal duration of antibody incubation was overnight at room temperature for all markers.

Post-analytic phase included comparison of expression profiles for selected markers in the Human Protein Atlas (HPA) database [1] with our staining in different mouse and human tissues. Further, we observed that antibodies developed against same protein had different frequency and intensities in same tissue. In addition, due to absence of availability of antibodies from same manufacturer as that used in HPA, it was crucial to identify control tissues using accessible antibodies.
Thus, expression controls and reference tissues selected for each marker included -

(i) TCF21 (cardiac myocytes, stromal cells of ovary, hepatocytes and germinal basal cells of testis),
(ii) E-cadherin (cardiac myocytes, hepatocytes, epithelial cells of small intestine and epithelial cells of prostate),
(iii) PARP1 (mucosa of small intestine, cardiac myocytes, and germinal basal cells of testis),
(iv) Slug (cardiac myocytes, somatic muscle of appendix, lymphocytes present in small intestine),
(v) ANXA2 (cardiac myocytes, somatic muscle of small intestine, epithelial and stromal cells of gall bladder) and,
(vi) HA (cartilage and small intestine).

The subjectivity of interpretation in the above analyses was minimized by consultation with experienced panel of pathologists.

References

1. Uhlen, M.; Fagerberg, L.; Hallstrom, B.M.; Lindskog, C.; Oksvold, P.; Mardinoglu, A.; Sivertsson, A.; Kampf, C.; Sjostedt, E.; Asplund, A.; et al. Tissue-based map of the human proteome. *Science (80).* **2015**, *347*, 1260419–1260419.

[I] <u>SOP for TCF21</u>

1. Introduction

TCF21 encodes a transcription factor of the basic helix-loop-helix family. TCF21 protein is mesoderm specific, and expressed in embryonic epicardium, mesenchyme-derived tissues of lung, gut, gonad, and mesenchymal as well as glomerular epithelial cells in kidney. Epigenetic alterations of *TCF21*are associated with head and neck squamous cell carcinoma (HNSCC) [1]. The epigenetic silencing of TCF21 by CpG island (CGI) hypermethylation is reported [2-4]; thus its methylation status has been proposed as diagnostic biomarker forurological and non-small cell lung cancers [4,5]. Down regulation of *TCF21* induces cell proliferation, migration and invasion in colorectal cancer [6] and leads to large sized tumors along with lymph node metastasis [7], while its over-expression inhibits cellular proliferation and migration [3,8]. The procedures described here were developed for detection of TCF21 protein by IHC in Formalin Fixed, Paraffin Embedded (FFPE) tissues and observed using bright microscopy.

2. Aim

To visualize TCF21 protein through IHC using TCF21-specific primary antibody and enzyme-conjugated secondary antibody.

3. Scope

This procedure applies to IHC-based detection of TCF21 in FFPE tissues.

4. Principle

The primary antibody binds to TCF21 protein if present in the specimen. Unbound antibody is removed by washing and peroxidase conjugated secondary antibody is added that reacts with tissue-bound primary antibody. Unbound antibody is again removed by washing and the tissue is incubated with freshly prepared chromogenic development reagent, 3, 3' diaminobenzidine (DAB Substrate), which reacts with peroxidase conjugated secondary antibody complex. Horse Radish-Peroxidase (HRP) activity on chromogenic substrate leads to deposit of brown insoluble precipitate at the antigenic sites containing primary antibody-specific epitopes [9].

5. Specimens

FFPE tissue cut at 5μm and fixed on poly-L-lysine (PLL) coated slide by drying at 60°C for at least 1hr in oven. Germinal cells of testis and heart tissue were selected as reference positive and negative expression control tissues respectively based on information from the Human Protein Atlas.

6. Protocol/Procedure

Materials

1. Coplin Jars for Staining
2. Graduated Cylinders
3. Pipettes and disposable tips
4. 1.5ml centrifuge tubes for solution preparation
5. Humidity chamber

6. Tissue papers
7. Slides
8. Cover slips

Equipment

1. Oven
2. De-cloaking chamber for antigen retrieval
3. Microscope

Reagents & Chemicals

1. TCF21 Rabbit polyclonal antibody raised against TCF21 of human origin (#ab32981, Abcam,Cambridge, MA, USA).
2. Secondary Conjugate (anti-rabbit) linked to HRP (#111-035-144, Jackson ImmunoResearch Laboratories, Inc.West Grove, PA, USA)
3. TBS pH 7.2-7.4 (#ML029, Himedia, Mumbai, India)
4. Hydrogen peroxide (#18755, Qualigens, Waltham, Massachusetts, USA)
5. Distilled water
6. Protein block (#HK085-5KE, Biogenex,Fremont, CA, USA)
7. Antigen retrieval buffer, Citrate Buffer, pH 6.1 (#ML089, Himedia, Mumbai, India)
8. Harris's hematoxylin(prepared in-house)
9. DAB (#34065, Thermo Pierce,Waltham, Massachusetts, USA)
10. DPX (#18404, Qualigens, Waltham, Massachusetts, USA)

7. Flowchart

Heat slide in oven or spirit lamp (5min) and Transfer in xylene solution (2x5min)

↓

Hydrate the slides by alcohol gradient
(100% EtOH: 2 x 5min →95% EtOH: 5min →70% EtOH: 5min → 50% EtOH: 5min →Running water 10min)

↓

Heat the decloaking chamber with Antigen Retrieval Buffer at 95-98ºC

↓

Transfer slides into pre-heated Antigen retrieval buffer at 95-98ºC (30min)

↓

Perform hydrogen peroxide block using 3% H_2O_2 in Distilled water (30-45min)

↓

Wash with distilled water (5min)

↓

Wash with 1xTris buffered saline (TBS pH7.2-7.4) (5min)

↓

Perform Protein Block (10min)

↓

Wash off excess protein block and give washes with TBS (twice)

↓

Apply primary antibody and incubate at Room Temperature (overnight)

↓

Wash with 1xTBS twice (5min)

↓

Apply secondary antibody conjugated to HRP (60min)

↓

Wash with 1xTBS (5min)

↓

Apply freshly prepared DAB

↓

Observe for color development under microscope with maximum duration of 10min

↓

Stop reaction by putting the slide in distilled water

↓

Counter stain with Hematoxylin (1-2min)

↓

Allow hematoxylin color to develop in running water or differentiate in alkaline solution

↓

Dehydrate in alcohol gradient
50% EtOH: 5 min →70% EtOH: 5 min →95% EtOH: 5 min → 100% EtOH: 2 x 5 min

↓

Wash in xylene (2 x 5min) and Mount cover slips using mounting media (DPX)

8. **Interpretation**
Presence of TCF21 imparts nuclear/cytoplasm staining (brown) to the normal cells. In the tissues lacking staining, absence of brown coloration with nuclei stained blue by counterstain hematoxylin is observed.

9. **Representative Quality Control**

Control Tissues	
Positive-<br>TESTIS	Negative-<br>MYOCARDIUM
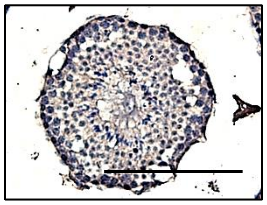	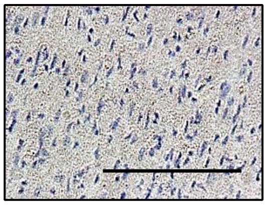

Figure: Microphotographs of TCF21 positive (testis, left) and negative (myocardium, right), which were considered as reference tissues. Scale bar is 100µm.

References

1. Weiss, D.; Stockmann, C.; Schrödter, K.; Rudack, C. Protein expression and promoter methylation of the candidate biomarker TCF21 in head and neck squamous cell carcinoma. Cell. Oncol. (Dordr). **2013**, 36, 213–24.
2. Tessema, M.; Willink, R.; Do, K.; Yu, Y.Y.; Yu, W.; Machida, E.O.; Brock, M.; Van Neste, L.; Stidley, C.A.; Baylin, S.B.; et al. Promoter methylation of genes in and around the candidate lung cancer susceptibility locus 6q23-25. Cancer Res. **2008**, 68, 1707–14.
3. Arab, K.; Smith, L.T.; Gast, A.; Weichenhan, D.; Huang, J.P.-H.; Claus, R.; Hielscher, T.; Espinosa, A. V; Ringel, M.D.; Morrison, C.D.; et al. Epigenetic deregulation of TCF21 inhibits metastasis suppressor KISS1 in metastatic melanoma. Carcinogenesis **2011**, 32, 1467–73.

4. Richards, K.L.; Zhang, B.; Sun, M.; Dong, W.; Churchill, J.; Bachinski, L.L.; Wilson, C.D.; Baggerly, K.A.; Yin, G.; Hayes, D.N.; et al. Methylation of the candidate biomarker TCF21 is very frequent across a spectrum of early-stage nonsmall cell lung cancers. Cancer **2011**, 117, 606–17.
5. Costa, V.L.; Henrique, R.; Danielsen, S.A.; Eknaes, M.; Patrício, P.; Morais, A.; Oliveira, J.; Lothe, R.A.; Teixeira, M.R.; Lind, G.E.; et al. TCF21 and PCDH17 methylation: An innovative panel of biomarkers for a simultaneous detection of urological cancers. Epigenetics **2011**, 6, 1120–30.
6. Dai, Y.; Duan, H.; Duan, C.; Zhou, R.; He, Y.; Tu, Q.; Shen, L. Down-regulation of TCF21 by hypermethylation induces cell proliferation, migration and invasion in colorectal cancer. Biochem. Biophys. Res. Commun. **2015**, 469, 430–6.
7. Wang, J.; Gao, X.; Wang, M.; Zhang, J. Clinicopathological significance and biological role of TCF21 mRNA in breast cancer. Tumour Biol. **2015**, 36, 8679–83.
8. Tan, J.; Zhang, G.; Liu, R.; Zhou, M.; Li, Z.; Wu, Z. Over-expression of transcription factor 21 inhibits the proliferation and migration and promotes apoptosis of SMMC-7721 cells. Xi Bao Yu Fen Zi Mian Yi Xue Za Zhi **2015**, 31, 884–8.
9. Dabbs, D.J. Diagnostic Immunohistochemistry, 4th Edition: Dabbs; 4th ed.; **2014**; ISBN 978-1-4557-4461-9.

[II] SOP for E-cadherin

1. **Introduction**

E-cadherin is a cell-cell adhesion glycoprotein considered to be a tumor suppressor in various epithelial malignancies like melanoma, hepatocellular carcinoma, head and neck [1]. Loss of E-cadherin function increases tumor cell proliferation, invasion, and metastasis and is associated with cancer progression [2]. On the contrary, its re-introduction in cell lines lacking E-cadherin expression causes change in phenotype from poorly differentiated to well differentiated [3]. E-cadherin is also a well known downstream target of Epithelial-to-Mesenchymal Transcription Factors (EMT-TFs) including Snail, Slug, Zeb1, Zeb2 and Twist1 [4-7]. In ovarian cancer patients, loss of E-cadherin relates to low Overall Survival [8,9]. The procedures described here were developed for detection of E-cadherin protein by IHC in FFPE tissues and observed using bright microscopy.

2. **Aim**

To visualize E-cadherin protein through IHC with use of E-cadherin-specific primary antibody and enzyme-conjugated secondary antibody.

3. **Scope**

This procedure applies to IHC for detection of E-cadherin in the FFPE tissues.

4. **Principle**

The primary antibody binds to E-cadherin protein if present in the specimen. Unbound antibody is removed by washing and peroxidase conjugated secondary antibody is added that reacts with tissue-bound primary antibody. Unbound antibody is again removed by washing and the tissue is incubated with freshly prepared chromogenic development reagent, 3, 3' diaminobenzidine (DAB Substrate), which reacts with peroxidase conjugated secondary antibody complex. Horse Radish-Peroxidase (HRP) activity on chromogenic substrate results in deposit of brown insoluble precipitate at the antigenic sites containing primary antibody-specific epitopes [10].

5. **Specimens**

FFPE tissue cut at 5µm and fixed on poly-L-lysine (PLL) coated slide by drying at 60°C for at least 1hr in oven. Liver and heart tissue were selected as reference positive and negative expression control tissues respectively based on information fromthe Human Protein Atlas.

6. **Protocol/Procedure**

Materials

1. Coplin Jars for Staining
2. Graduated Cylinders
3. Pipettes and disposable tips
4. 1.5ml centrifuge tubes for solution preparation
5. Humidity chamber
6. Tissue papers
7. Slides
8. Cover slips

Equipment

1. Oven
2. Decloaking chamber for antigen retrieval
3. Microscope

Reagents & Chemicals

1. E-cadherin Mouse monoclonal antibody raised against E-cadherin of human origin (#AM390-5M, Biogenex,Fremont, CA, USA).
2. Secondary Conjugate (anti-mouse) linked to HRP (#715-035-150, Jackson Immuno Research Laboratories, Inc.West Grove, PA, USA)
3. TBS pH 7.2-7.4 (#ML029, Himedia, Mumbai, India)
4. Hydrogen peroxide (#18755, Qualigens,Waltham, Massachusetts, USA)
5. Distilled water
6. Protein block (#HK085-5KE, Biogenex,Fremont, CA, USA)
7. Antigen retrieval buffer, Tris-EDTA Buffer, pH 9.0 (#ML087, Himedia, Mumbai, India)
8. Harris's hematoxylin(prepared in-house)
9. DAB (#34065, Thermo Pierce,Waltham, Massachusetts, USA)
10. DPX (#18404, Qualigens,Waltham, Massachusetts, USA)

7. Flow chart

Heat slide in oven or spirit lamp (5min) and transfer in xylene solution (2x5min)

↓

Hydrate the slides by alcohol gradient.

(100% EtOH: 2 x 5 min→95% EtOH: 5 min→70% EtOH: 5 min→ 50% EtOH: 5 min→Running water 10 min)

↓

Heat the decloaking chamber with Antigen Retrieval Buffer at 95-98ºC.

↓

Transfer slides into pre-heated Antigen retrieval buffer at 95-98ºC (30min).

↓

Perform hydrogen peroxide block using 3% H_2O_2 in Distilled water (60min).

↓

Wash with distilled water (5min).

↓

Wash with 1xTris buffered saline (TBS pH7.2-7.4) (5min).

↓

Perform Protein Block (10min).

↓

Wash off excess protein block and give washes with TBS (twice).

↓

Apply primary antibody and incubate at Room Temperature (overnight).

↓

Wash with 1xTBS twice (5min).

↓

Apply conjugate (60min).

↓

Wash with 1xTBS (5min).

↓

Apply freshly prepared DAB.

↓

Observe for color development under microscope with maximum duration of 10min.

↓

Stop reaction by putting the slide in distilled water.

↓

Counter stain with Hematoxylin (1-2min).

↓

Allow Hematoxylin color to develop in running water or differentiate in alkaline solution.

↓

Dehydrate in alcohol gradient.

50% EtOH: 5 min→70% EtOH: 5 min→95% EtOH: 5 min→ 100% EtOH: 2 x 5 min.

↓

Wash in xylene (2 x 5min) and Mount cover slips using mounting media (DPX).

8. **Interpretation**
   Presence of E-cadherin imparts brown color to the cell membrane. In tissues lacking staining, absence of brown coloration with nuclei stained blue by the counter stain Hematoxylin is observed.

9. **Representative Quality Control (IHC)**

Control Tissues	
Positive-<br>LIVER	Negative-<br>MYOCARDIUM
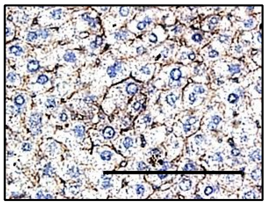	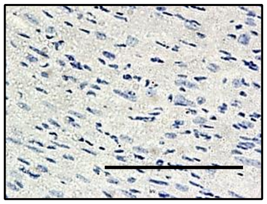

Figure: Microphotographs of E-cadherin positive (liver, left) and negative (myocardium, right) tissues. Scale bar is 100μm.

References

1. Berx, G.; van Roy, F. Involvement of Members of the Cadherin Superfamily in Cancer. Cold Spring Harb. Perspect. Biol. **2009**, 1, a003129–a003129.
2. Rodriguez, F.J.; Lewis-Tuffin, L.J.; Anastasiadis, P.Z. E-cadherin's dark side: possible role in tumor progression. Biochim. Biophys. Acta **2012**, 1826, 23–31.
3. Wong, A.S.T.; Gumbiner, B.M. Adhesion-independent mechanism for suppression of tumor cell invasion by E-cadherin. J. Cell Biol. **2003**, 161, 1191–203.
4. Cano, A.; Pérez-Moreno, M.A.; Rodrigo, I.; Locascio, A.; Blanco, M.J.; del Barrio, M.G.; Portillo, F.; Nieto, M.A. The transcription factor snail controls epithelial-mesenchymal transitions by repressing E-cadherin expression. Nat. Cell Biol. **2000**, 2, 76–83.
5. Batlle, E.; Sancho, E.; Francí, C.; Domínguez, D.; Monfar, M.; Baulida, J.; García De Herreros, A. The transcription factor snail is a repressor of E-cadherin gene expression in epithelial tumour cells. Nat. Cell Biol. **2000**, 2, 84–9.
6. Bolós, V.; Peinado, H.; Pérez-Moreno, M.A.; Fraga, M.F.; Esteller, M.; Cano, A. The transcription factor Slug represses E-cadherin expression and induces epithelial to mesenchymal transitions: a comparison with Snail and E47 repressors. J. Cell Sci. **2003**, 116, 499–511.
7. De Craene, B.; Berx, G. Regulatory networks defining EMT during cancer initiation and progression. Nat. Rev. Cancer **2013**, 13, 97–110.
8. Peng, H.-L.; He, L.; Zhao, X. Association of reduced immunohistochemical expression of E-cadherin with a poor ovarian cancer prognosis--results of a meta-analysis. Asian Pac. J. Cancer Prev. **2012**, 13, 2003–7.
9. Bačić, B.; Haller, H.; Mrklić, I.; Košta, V.; Čarić, A.; Tomić, S. Prognostic role of E-cadherin in patients with advanced serous ovarian cancer. Arch. Gynecol. Obstet. **2013**, 287, 1219–24.
10. Dabbs, D.J. Diagnostic Immunohistochemistry, 4th Edition: Dabbs; 4th ed.; 2014; ISBN 978-1-4557-4461-9.

[III] SOP for PARP1

1. **Introduction**

Poly [ADP-ribose] polymerase 1 (PARP1) is a nuclear enzyme encoded by *PARP1*, which is engaged in the repair of DNA single-strand breaks *via* base excision repair pathway and also plays role in regulation of transcription and cell cycle progression [1]. PARP1 activity is generally high in tumor cells with defects in homologous recombination, such as *BRCA1* & *BRCA2* mutation associated ovarian and breast cancers [2,3]. Association of *BRCA1/2*mutations and PARP1 expression through IHC has been studied in ovarian cancer [4,5]. Inhibition of PARP activity can improve the therapeutic index of chemotherapy in cases where the DNA damage is discriminatory *i.e.* more effective in tumor than normal cells, since two concurrent non-functional DNA damage repair proteins lead to 'synthetic lethality', preventing tumor cells to tolerate further DNA damage [6]. This knowledge is exploited pharmacologically by using PARP inhibitors that imitate nicotinamide moiety of Nicotinamide Adenine Dinucleotide (NAD) and upon binding to catalytic domain of PARP prevent further changes in it, thereby releasing PARP from the DNA [7]. The procedures described here were developed for detection of PARP1 protein by IHC in formalin fixed, paraffin embedded(FFPE) tissues and observed using bright microscopy.

2. **Aim**

To visualize PARP1 protein through IHC with use of PARP1-specific primary antibody and enzyme-conjugated secondary antibody.

3. **Scope**

This procedure applies to IHC for detection of PARP1 in the FFPE tissues.

4. **Principle**

The primary antibody binds to PARP1 protein if present in the specimen. Unbound antibody is removed by washing and peroxidase conjugated secondary antibody is added that reacts with tissue-bound primary antibody. Unbound antibody is again removed by washing and the tissue is incubated with freshly prepared chromogenic development reagent, 3, 3' diaminobenzidine (DAB Substrate), which reacts with peroxidase conjugated secondary antibody complex. Horse Radish-Peroxidase (HRP) activity on chromogenic substrate results in deposit of brown insoluble precipitate at the antigenic sites containing primary antibody-specific epitopes [8].

5. **Specimens**

FFPE tissue cut at 5μm and fixed on poly-L-lysine (PLL) coated slide by drying at 60°C for at least 1hr in oven. Germinal cells of testis and mucosa of small intestine were selected as reference positive and negative expression control tissues respectively based on information fromthe Human Protein Atlas.

6. **Protocol/Procedure**

Materials

1. Coplin Jars for Staining
2. Graduated Cylinders
3. Pipettes and disposable tips
4. 1.5ml centrifuge tubes for solution preparation

5. Humidity chamber
6. Tissue papers
7. Slides
8. Cover slips

Equipment
1. Oven
2. Decloaking chamber for antigen retrieval
3. Microscope

Reagents & Chemicals
1. PARP1 (H-300) Rabbit polyclonal antibody raised against PARP of human origin (#sc-25780, Santa Cruz Biotechnology, Inc. Dallas, Texas, U.S.A.)
2. Secondary Conjugate (anti-rabbit) linked to HRP (#111-035-144, Jackson Immuno Research Laboratories, Inc.West Grove, PA, USA)
3. TBS pH 7.2-7.4 (#ML029, Himedia, Mumbai, India)
4. Hydrogen peroxide (#18755, Qualigens,Waltham, Massachusetts, USA)
5. Distilled water
6. Protein block (#HK085-5KE, Biogenex,Fremont, CA, USA)
7. Antigen retrieval buffer, Tris EDTA Buffer, pH 9.0 (#ML087, Himedia, Mumbai, India)
8. Harris' hematoxylin(prepared in-house)
9. DAB (#34065, Thermo Pierce,Waltham, Massachusetts, USA)
10. DPX (#18404, Qualigens,Waltham, Massachusetts, USA)

7. **Flow chart**

Heat slide in oven or spirit lamp (5min) and transfer in xylene solution (2x5min)

↓

Hydrate the slides by alcohol gradient.
(100% EtOH: 2 x 5 min→95% EtOH: 5 min→70% EtOH: 5 min→ 50% EtOH: 5 min→Running water 10 min)

↓

Heat the decloaking chamber with Antigen Retrieval Buffer at 95-98ºC.

↓

Transfer slides into pre-heated Antigen Retrieval Buffer at 95-98ºC (30min).

↓

Perform hydrogen peroxide block using 3% H_2O_2 in Distilled water (60min).

↓

Wash with distilled water (5min).

↓

Wash with 1xTris buffered saline (TBS pH7.2-7.4) (5min).

↓

Perform Protein Block (10min).

↓

Wash off excess protein block and give washes with TBS (twice).

↓

Apply primary antibody and incubate at Room Temperature (overnight).

↓

Wash with 1xTBS twice (5min).

↓

Apply conjugate (60min).

↓

Wash with 1xTBS (5min).

↓

Apply freshly prepared DAB.

↓

Observe for color development under microscope with maximum duration of 10min.

↓

Stop reaction by putting the slide in distilled water.

↓

Counter stain with Hematoxylin (1-2min).

↓

Allow hematoxylin color to develop in running water or differentiate in alkaline solution.

↓

Dehydrate in alcohol gradient.
50% EtOH: 5 min→70% EtOH: 5 min→95% EtOH: 5 min→ 100% EtOH: 2 x 5 min.

↓

Wash in xylene (2 x 5min) and Mount cover slips using mounting media (DPX).

8. **Interpretation**

Presence of PARP1 imparts nuclear staining (brown) to the normal cells. In the tissues lacking staining, absence of brown coloration with nuclei stained blue by the counter stain hematoxylin is observed.

9. **Representative Quality Control**

Control Tissues	
Positive-<br>TESTIS	Negative-<br>Mucosa of Small Intestine
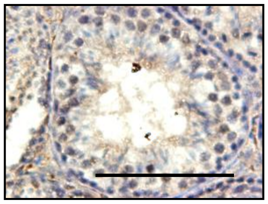	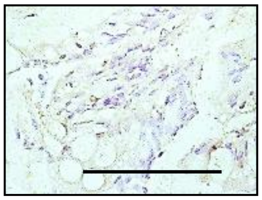

Figure: Microphotographs of PARP1 positive (testis, left) and negative (Mucosa of Small Intestine, right) tissues. Scale bar is 100μm.

References

1. Iyama, T.; Wilson, D.M. DNA repair mechanisms in dividing and non-dividing cells. *DNA Repair (Amst).* **2013**, *12*, 620–636.
2. Ossovskaya, V.; Koo, I.C.; Kaldjian, E.P.; Alvares, C.; Sherman, B.M. Upregulation of Poly (ADP-Ribose) Polymerase-1 (PARP1) in Triple-Negative Breast Cancer and Other Primary Human Tumor Types. *Genes Cancer* **2010**, *1*, 812–21.
3. Chow, J.P.H.; Man, W.Y.; Mao, M.; Chen, H.; Cheung, F.; Nicholls, J.; Tsao, S.W.; Li Lung, M.; Poon, R.Y.C. PARP1 is overexpressed in nasopharyngeal carcinoma and its inhibition enhances radiotherapy. *Mol. Cancer Ther.* **2013**, *12*, 2517–28.
4. Marques, M.; Beauchamp, M.-C.; Fleury, H.; Laskov, I.; Qiang, S.; Pelmus, M.; Provencher, D.; Mes-Masson, A.-M.; Gotlieb, W.H.; Witcher, M. Chemotherapy reduces PARP1 in cancers of the ovary: implications for future clinical trials involving PARP inhibitors. *BMC Med.* **2015**, *13*, 217.
5. Magdalena, K.; Monika, Z.; Adam, G.; Magdalena, R.; Marzena, L.; Wojciech, B.; Janusz, L.; Bartosz, W. Detection of somatic BRCA1/2 mutations in ovarian cancer - next-generation sequencing analysis of 100 cases. *Cancer Med.* **2016**, *5*, 1640–6.
6. Iglehart, J.D.; Silver, D.P. Synthetic Lethality — A New Direction in Cancer-Drug Development. *N. Engl. J. Med.* **2009**, *361*, 189–191.
7. Kummar, S.; Chen, A.; Parchment, R.E.; Kinders, R.J.; Ji, J.; Tomaszewski, J.E.; Doroshow, J.H. Advances in using PARP inhibitors to treat cancer. *BMC Med.* **2012**, *10*, 25.
8. Dabbs, D.J. *Diagnostic Immunohistochemistry, 4th Edition: Dabbs*; 4th ed.; 2014; ISBN 978-1-4557-4461-9.

[IV] SOP for Slug

1. Introduction

Slug (encoded by *SNAI2*) is a transcriptional repressor binding to E-box motifs involved in epithelial-mesenchymal transition and radio- and chemo-resistance activity [1,2]. It is reported to be overexpressed in cancers of ovary, stomach, lung, colorectum, brain, breast, prostate, liver among others [2-9]. Knockdown of Slug by lentivirus-mediated shRNA or RNAi inhibits cellular proliferation and invasion properties in colorectal and lung cancer cells [10,11]. Interestingly, Slug expression has been reported to be reduced in post neoadjuvant chemotherapy (NACT) in patients of breast cancer [12]. Prostate cancer patients given combinatorial treatment of mTOR/Erk/HSP90 inhibitors led to inhibition of metastatic capability *via* Slug inhibition [13]. The procedures described here were developed for detection of Slug protein by IHC in formalin fixed, paraffin embedded (FFPE) tissues and observed using bright microscopy.

2. Aim

To visualize Slug protein through IHC with use of Slug-specific primary antibody and enzyme-conjugated secondary antibody.

3. Scope

This procedure applies to IHC for detection of Slug in the FFPE tissues.

4. Principle

The primary antibody binds to Slug protein if present in the specimen. Unbound antibody is removed by washing and peroxidase conjugated secondary antibody is added that reacts with tissue-bound primary antibody. Unbound antibody is again removed by washing and the tissue is incubated with freshly prepared chromogenic development reagent, 3, 3' diaminobenzidine (DAB Substrate), which reacts with peroxidase conjugated secondary antibody complex. Horse Radish-Peroxidase (HRP) activity on chromogenic substrate results in deposit of brown insoluble precipitate at the antigenic sites containing primary antibody-specific epitopes [14].

5. Specimens

FFPE tissue cut at 5μm and fixed on poly-L-lysine (PLL) coated slide by drying at 60°C for at least 1hr in oven. Germinal cells of testis and heart tissue were selected as reference positive and negative expression control tissues respectively based on information from the Human Protein Atlas.

6. Protocol/Procedure

Materials

1. Coplin Jars for staining
2. Graduated cylinders
3. Pipettes and disposable tips
4. 1.5ml centrifuge tubes for solution preparation
5. Humidity chamber
6. Tissue papers
7. Slides

8. Cover slips

Equipment

1. Oven
2. Decloaking chamber for antigen retrieval
3. Microscope

Reagents & Chemicals

1. Slug Rabbit polyclonal antibody raised against Slug of human origin (#ab27568, Abcam,Cambridge, MA, USA).
2. Secondary Conjugate (anti-rabbit) linked to HRP (#111-035-144, Jackson Immuno Research Laboratories, Inc.West Grove, PA, USA)
3. TBS pH 7.2-7.4 (#ML029, Himedia, Mumbai, India)
4. Hydrogen peroxide (#18755, Qualigens, Waltham, Massachusetts, USA)
5. Distilled water
6. Protein block (#HK085-5KE, Biogenex,Fremont, CA, USA)
7. Antigen retrieval buffer, Citrate Buffer, pH 6.1 (#ML089, Himedia, Mumbai, India)
8. Harris's hematoxylin(prepared in-house)
9. DAB (#34065, Thermo Pierce,Waltham, Massachusetts, USA)
10. DPX (#18404, Qualigens, Waltham, Massachusetts, USA)

7. **Flow chart**

Heat slide in oven or spirit lamp (5min) and transfer in xylene solution (2x5min)

↓

Hydrate the slides by alcohol gradient

(100% EtOH: 2 x 5 min→95% EtOH: 5 min→70% EtOH: 5 min→ 50% EtOH: 5 min→Running water 10 min)

↓

Heat the decloaking chamber with Antigen Retrieval Buffer at 95-98ºC

↓

Transfer slides into pre-heated Antigen Retrieval Buffer at 95-98ºC (30min)

↓

Perform hydrogen peroxide block using 3% H_2O_2 in distilled water (60min)

↓

Wash with distilled water (5min)

↓

Wash with 1xTris buffered saline (TBS pH7.2-7.4) (5min)

↓

Perform Protein Block (10min)

↓

Wash off excess protein block and give washes with TBS (twice)

↓

Apply primary antibody and incubate at Room Temperature (overnight)

↓

Wash with 1xTBS twice (5min)

↓

Apply conjugate (60min)

↓

Wash with 1xTBS (5min)

↓

Apply freshly prepared DAB

↓

Observe for color development under microscope with maximum duration of 10min

↓

Stop reaction by putting the slide in distilled water

↓

Counterstain with Harris's Hematoxylin (1-2min)

↓

Allow Hematoxylin color to develop in running water or differentiate in alkaline solution

↓

Dehydrate in alcohol gradient

50% EtOH: 5 min→70% EtOH: 5 min→95% EtOH: 5 min→ 100% EtOH: 2 x 5 min

↓

Wash in xylene (2 x 5min) and Mount cover slips using mounting media (DPX)

8. Interpretation

Presence of Slug imparts nuclear/cytoplasmic staining (brown) to the normal cells. In the tissues lacking staining, absence of brown coloration with nuclei stained blue by the counter stain Hematoxylin is observed.

9. Representative Quality Control

Control Tissues	
Positive-<br>TESTIS	Negative-<br>Heart
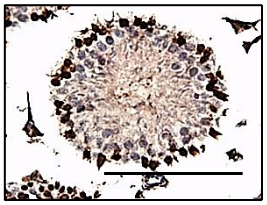	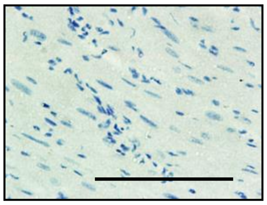

Figure: Microphotographs of Slug positive (testis, left) and negative (myocardium, right) tissues. Scale bar is 100μm.

References

1. Kurrey, N.K.; K, A.; Bapat, S.A. Snail and Slug are major determinants of ovarian cancer invasiveness at the transcription level. *Gynecol. Oncol.* **2005**, *97*, 155–65.
2. Kurrey, N.K.; Jalgaonkar, S.P.; Joglekar, A. V; Ghanate, A.D.; Chaskar, P.D.; Doiphode, R.Y.; Bapat, S.A. Snail and slug mediate radioresistance and chemoresistance by antagonizing p53-mediated apoptosis and acquiring a stem-like phenotype in ovarian cancer cells. *Stem Cells* **2009**, *27*, 2059–68.
3. Castro Alves, C.; Rosivatz, E.; Schott, C.; Hollweck, R.; Becker, I.; Sarbia, M.; Carneiro, F.; Becker, K.-F. Slug is overexpressed in gastric carcinomas and may act synergistically with SIP1 and Snail in the down-regulation of E-cadherin. *J. Pathol.* **2007**, *211*, 507–15.
4. Shih, J.-Y.; Yang, P.-C. The EMT regulator slug and lung carcinogenesis. *Carcinogenesis* **2011**, *32*, 1299–304.
5. Shioiri, M.; Shida, T.; Koda, K.; Oda, K.; Seike, K.; Nishimura, M.; Takano, S.; Miyazaki, M. Slug expression is an independent prognostic parameter for poor survival in colorectal carcinoma patients. *Br. J. Cancer* **2006**, *94*, 1816–22.
6. Yang, H.W.; Menon, L.G.; Black, P.M.; Carroll, R.S.; Johnson, M.D. SNAI2/Slug promotes growth and invasion in human gliomas. *BMC Cancer* **2010**, *10*, 301.
7. Elloul, S.; Elstrand, M.B.; Nesland, J.M.; Tropé, C.G.; Kvalheim, G.; Goldberg, I.; Reich, R.; Davidson, B. Snail, Slug, and Smad-interacting protein 1 as novel parameters of disease aggressiveness in metastatic ovarian and breast carcinoma. *Cancer* **2005**, *103*, 1631–43.
8. Uygur, B.; Wu, W.-S. SLUG promotes prostate cancer cell migration and invasion via CXCR4/CXCL12 axis. *Mol. Cancer* **2011**, *10*, 139.
9. Giannelli, G.; Bergamini, C.; Fransvea, E.; Sgarra, C.; Antonaci, S. Laminin-5 with transforming growth factor-beta1 induces epithelial to mesenchymal transition in hepatocellular carcinoma. *Gastroenterology* **2005**, *129*, 1375–83.
10. Wang, Y.-P.; Wang, M.-Z.; Luo, Y.-R.; Shen, Y.; Wei, Z.-X. Lentivirus-mediated shRNA interference targeting SLUG inhibits lung cancer growth and metastasis. *Asian Pac. J. Cancer Prev.* **2012**, *13*, 4947–51.
11. Qian, J.; Liu, H.; Chen, W.; Wen, K.; Lu, W.; Huang, C.; Fu, Z. Knockdown of Slug by RNAi inhibits the proliferation and invasion of HCT116 colorectal cancer cells. *Mol. Med. Rep.* **2013**, *8*, 1055–9.
12. Riemenschnitter, C.; Teleki, I.; Tischler, V.; Guo, W.; Varga, Z. Stability and prognostic value of Slug, Sox9 and Sox10 expression in breast cancers treated with neoadjuvant chemotherapy. *Springerplus* **2013**, *2*, 695.
13. Ding, G.; Feng, C.; Jiang, H.; Ding, Q.; Zhang, L.; Na, R.; Xu, H.; Liu, J. Combination of rapamycin, CI-1040, and 17-AAG inhibits metastatic capacity of prostate cancer via Slug inhibition. *PLoS One* **2013**, *8*, e77400.
14. Dabbs, D.J. *Diagnostic Immunohistochemistry, 4th Edition: Dabbs*; 4th ed.; 2014; ISBN 978-1-4557-4461-9.

[V] <u>AnnexinA2 (ANXA2)</u>

1. **Introduction**

AnnexinA2, a member of Annexin family, isa calcium-binding protein usually present at the extracellular surface of endothelial cells and certain tumors [1,2]. High ANXA2 expression is associated with cancers of ovary, breast, prostate, liver and pancreas [3-7]. ANXA2 also participates in tumor cell metastases [8,4,9]. Further, inhibition of ANXA2 in nasopharyngeal carcinoma (NPC) by shRNA led to suppression of cellular proliferation, cell migration, invasion and vascular formation [10]. A monoclonal antibody named mAb150 was developed against ANXA2 in our lab. The procedures described here were developed for detection of ANXA2 protein by IHC using mAb150 in formalin fixed, paraffin embedded(FFPE) tissues and observed using bright microscopy.

2. **Aim**

To visualize ANXA2 protein through IHC with use of ANXA2-specific in-house developed primary antibody mAb150 and enzyme-conjugated secondary antibody.

3. **Scope**

This procedure applies to IHC for detection of ANXA2in the FFPE tissues.

4. **Principle**

The primary antibody, mAb150 binds to ANXA2 protein if present in the specimen. Unbound antibody is removed by washing and peroxidase conjugated secondary antibody is added that reacts with tissue-bound primary antibody. Unbound antibody is again removed by washing and the tissue is incubated with freshly prepared chromogenic development reagent, 3, 3' diaminobenzidine (DAB Substrate), which reacts with peroxidase conjugated secondary antibody complex. Horse Radish-Peroxidase (HRP) activity on chromogenic substrate results in deposit of brown insoluble precipitate at the antigenic sites containing primary antibody-specific epitopes [11].

5. **Specimens**

FFPE tissue cut at 5μm and fixed on poly-L-lysine (PLL) coated slide by drying at 60°C for at least 1hr in oven. Epithelial cells of prostate and heart tissue were selected as reference positive and negative expression control tissues respectively based on information from the Human Protein Atlas.

6. **Protocol/Procedure**

Materials

1. Coplin Jars for Staining
2. Graduated Cylinders
3. Pipettes and disposable tips
4. 1.5ml centrifuge tubes for solution preparation
5. Humidity chamber
6. Tissue papers
7. Slides
8. Cover slips

Equipment

1. Oven
2. Decloaking chamber for antigen retrieval
3. Microscope

Reagents & Chemicals

1. mAb150 antibody raised against ANXA2 of human origin (in-house developed).
2. Secondary Conjugate (anti-mouse) linked to HRP (715-035-150, Jackson ImmunoResearch Laboratories, Inc.West Grove, PA, USA)
3. TBS pH 7.2-7.4 (ML029, Himedia, Mumbai, India)
4. Hydrogen peroxide (18755, Qualigens, Waltham, Massachusetts, USA)
5. Distilled water
6. Protein block (HK085-5KE, Biogenex,Fremont, CA, USA)
7. Antigen retrieval Citrate Buffer, pH 6.1 (ML089, Himedia, Mumbai, India)
8. Harris' hematoxylin(prepared in-house)
9. DAB (34065, Thermo Pierce, Waltham, Massachusetts, USA)
10. DPX (18404, Qualigens, Waltham, Massachusetts, USA)

7. **Flow chart**

Heat slide in oven or spirit lamp (5min) and transfer in xylene solution (2x5min)

↓

Hydrate the slides by alcohol gradient.

(100% EtOH: 2 x 5 min→95% EtOH: 5 min→70% EtOH: 5 min→ 50% EtOH: 5 min→Running water 10 min)

↓

Heat the decloaking chamber with Antigen Retrieval Buffer at 95-98ºC.

↓

Transfer slides into pre-heated Antigen Retrieval Buffer at 95-98ºC (30min).

↓

Perform hydrogen peroxide block using 3% H_2O_2 in Distilled water (60min).

↓

Wash with distilled water (5min).

↓

Wash with 1xTris buffered saline (TBS pH7.2-7.4) (5min).

↓

Perform Protein Block (10min).

↓

Wash off excess Protein Block and give washes with TBS (twice).

↓

Apply primary antibody and incubate at Room Temperature (overnight).

↓

Wash with 1xTBS twice (5min).

↓

Apply conjugate (60min) and wash with 1xTBS (5min).

↓

Apply freshly prepared DAB.

↓

Observe for color development under microscope with maximum duration of 10min.

↓

Stop reaction by putting the slide in distilled water.

↓

Counter stain with Hematoxylin (1-2min).

↓

Allow Hematoxylin color to develop in running water or differentiate in alkaline solution.

↓

Dehydrate in alcohol gradient.

50% EtOH: 5 min→70% EtOH: 5 min→95% EtOH: 5 min→ 100% EtOH: 2 x 5 min.

↓

Wash in xylene (2 x 5min) and mount cover slips using mounting media (DPX).

8. **Interpretation**

Presence of ANXA2 imparts nuclear/membrane-cytoplasmic staining (brown) to the normal cells. In the tissues lacking staining, absence of brown coloration with nuclei stained blue by the counter stain Hematoxylin is observed.

9. **Representative Quality Control**

Control Tissues	
Positive-<br>GALL BLADDER	Negative-<br>MYOCARDIUM
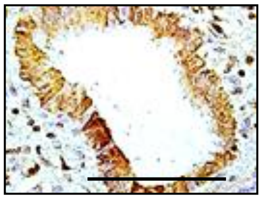	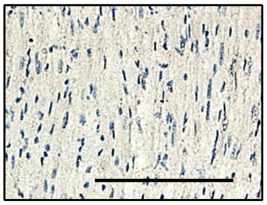

Figure: Microphotographs of ANXA2 positive (gall bladder, left) and negative (myocardium, right) tissues. Scale bar is 100µm.

References

1. Hajjar, K.A.; Guevara, C.A.; Lev, E.; Dowling, K.; Chacko, J. Interaction of the fibrinolytic receptor, annexin II, with the endothelial cell surface. Essential role of endonexin repeat 2. *J. Biol. Chem.* **1996**, *271*, 21652–9.
2. Mai, J.; Waisman, D.M.; Sloane, B.F. Cell surface complex of cathepsin B/annexin II tetramer in malignant progression. *Biochim. Biophys. Acta* **2000**, *1477*, 215–30.
3. Deng, Y.; Chen, C.; Hua, M.; Xi, Q; Liu, R, Yang, S; Liu, j; Zhong, j; Tang, M; Lu, S; Tang, C; Wang, Y. Annexin A2 plays a critical role in epithelial ovarian cancer. *Arch Gynecol Obstet.* **2014**, *292*(1):175-82.
4. Sharma, M.R.; Koltowski, L.; Ownbey, R.T.; Tuszynski, G.P.; Sharma, M.C. Angiogenesis-associated protein annexin II in breast cancer: Selective expression in invasive breast cancer and contribution to tumor invasion and progression. *Exp. Mol. Pathol.* **2006**, *81*, 146–156.
5. Inokuchi, J.; Narula, N.; Yee, D.S.; Skarecky, D.W.; Lau, A.; Ornstein, D.K.; Tyson, D.R. Annexin A2 positively contributes to the malignant phenotype and secretion of IL-6 in DU145 prostate cancer cells. *Int. J. Cancer* **2009**, *124*, 68–74.
6. Mohammad, H.S.; Kurokohchi, K.; Yoneyama, H.; Tokuda, M.; Morishita, A.; Jian, G.; Shi, L.; Murota, M.; Tani, J.; Kato, K.; et al. Annexin A2 expression and phosphorylation are up-regulated in hepatocellular carcinoma. *Int. J. Oncol.* **2008**, *33*, 1157–63.
7. Vishwanatha, J.K.; Chiang, Y.; Kumble, K.D.; Hollingsworth, M.A.; Pour, P.M. Enhanced expression of annexin II in human pancreatic carcinoma cells and primary pancreatic cancers. *Carcinogenesis* **1993**, *14*, 2575–2579.
8. Díaz, V.M.; Hurtado, M.; Thomson, T.M.; Reventós, J.; Paciucci, R. Specific interaction of tissue-type plasminogen activator (t-PA) with annexin II on the membrane of pancreatic cancer cells activates plasminogen and promotes invasion in vitro. *Gut* **2004**, *53*, 993–1000.
9. Shiozawa, Y.; Havens, A.M.; Jung, Y.; Ziegler, A.M.; Pedersen, E.A.; Wang, J.; Wang, J.; Lu, G.; Roodman, G.D.; Loberg, R.D.; et al. Annexin II/annexin II receptor axis regulates adhesion, migration, homing, and growth of prostate cancer. *J. Cell. Biochem.* **2008**, *105*, 370–380.
10. Chen, C.Y.; Lin, Y.S.; Chen, C.L.; Chao, P.Z.; Chiou, J.F.; Kuo, C.C.; Lee, F.P.; Lin, Y.F.; Sung, Y.H.; Lin, Y.T.; et al. Targeting annexin A2 reduces tumorigenesis and therapeutic resistance of nasopharyngeal carcinoma. *Oncotarget* **2015**, *6*.
11. Dabbs, D.J. *Diagnostic Immunohistochemistry, 4th Edition: Dabbs*; 4th ed.; 2014; ISBN 978-1-4557-4461-9.

[VI] SOP for Hyaluronic Acid

1. **Introduction**

Hyaluronic Acid (HA) or hyaluronan is a major non-proteoglycan polysaccharide component of extracellular matrix (ECM) essential for proper cell growth, organ structural stability and tissue organization [1]. Distribution of HA in the body depends upon structural integrity of the native tissue and physiological requirements. The extent of its presence in any tissue depends on its synthesis by the enzymes HA synthases [2]. Excess HA synthesis and accumulation occurs in pathologies like cardiovascular diseases [3,4], colorectal and breast cancer [5,6,3], *etc*. Excessive hyaluronan also upregulates EMT-transcription factors and promotes stem cell fate [7]. Specific targeting of a hyaluronan receptor *viz.* Cluster of Differentiation 44 (CD44) has been studied in ovarian cancer cells [8]. The procedures described here were developed for detection of hyaluronan fiber protein by histochemistry (HC) in formalin fixed, paraffin embedded (FFPE) tissues and observed using bright microscopy.

2. **Aim**

To visualize hyaluronic acid through histochemical analyses with use of Alcian blue stain and digestion by hyaluronidase enzyme.

3. **Scope**

This procedure applies to histochemistry for detection of hyaluronan in the FFPE tissues.

4. **Principle**

Some mesotheliomas have hyaluronan that can be stained with Alcian blue or colloidal iron. Mucins like chondroitin sulphates A and C and hyaluronan are digested with hyaluronidase. Loss of staining as compared to undigested consecutive section establishes presence of one or more of the three hyaluronidase labile mucins.

5. **Specimens**

FFPE tissue was cut at 5µm and fixed on poly-L-lysine coated slide by drying at 60°C for at least 1hr in oven. Reference expression control tissues were selected based on information from IHC World and on various tissues assayed. Positive expression tissue control used include small intestine and negative expression was not identified.

6. **Protocol/Procedure**

Materials

1. Coplin Jars for Staining
2. Graduated Cylinders
3. Pipettes and disposable tips
4. 1.5ml centrifuge tubes for solution preparation
5. Humidity chamber
6. Tissue papers
7. Slides
8. Cover slips

Equipment

1. Oven
2. 37°C incubator/oven for enzymatic digestion of HA
3. Microscope

Reagents & Chemicals

1. Hyaluronidase enzyme (H-3504, Sigma-Aldrich, Billerica, Massachusetts, USA)
2. Freshly prepared normal saline
3. Alcian blue 8GX (5500, Fluka, Billerica, Massachusetts, USA)
4. Nuclear Fast Red Solution (N3020, Sigma-Aldrich, Billerica, Massachusetts, USA)
5. Distilled water
6. DAB (34065, Thermo Pierce,Waltham, Massachusetts, USA)
7. DPX (18404, Qualigens,Waltham, Massachusetts, USA)

7. Flow chart

Heat slide in oven or spirit lamp to melt wax

↓

Transfer in xylene solution to dissolve wax

↓

Hydrate the slides by alcohol gradient

↓

Wash with distilled water (5min)

↓

Cover control and test section with phosphate buffer and hyaluronidase respectively (1hr at 37ºC)

↓

Wash in running water for 5min

↓

Apply Alcian blue stain and incubate for 1hr at Room Temperature

↓

Wash with running water for 5min

↓

Apply conjugate for 1hr

↓

Counter stain with Nuclear Fast Red Solution for 1-3min

↓

Dehydrate in alcohol gradient

↓

Wash in xylene for 5min

↓

Mount cover slips using mounting media (DPX)

8. **Interpretation**

Hyaluronic acid in the extracellular fibers/mucin residues is stained blue by Alcian blue in the enzymatic undigested tissue while hyaluronan is absent in the digested section. Mucins non-labile for hyaluronidase are observed as blue colored residues.

9. **Representative Quality Control (Histochemistry)**

Control Tissues	
Positive- Small Intestine	
Enzymatic untreated	Enzymatic treated
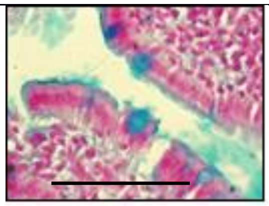	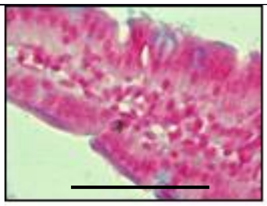

Figure: Microphotographs of hyaluronidase untreated(left) and treated (right) sections. Scale bar is 100µm.

References

1. Platt, V.M.; Szoka, F.C. Anticancer therapeutics: targeting macromolecules and nanocarriers to hyaluronan or CD44, a hyaluronan receptor. *Mol. Pharm.* **2008**, *5*, 474–86.
2. Weigel, P.H.; DeAngelis, P.L. Hyaluronan synthases: a decade-plus of novel glycosyltransferases. *J. Biol. Chem.* **2007**, *282*, 36777–81.
3. Toole, B.P.; Wight, T.N.; Tammi, M.I. Hyaluronan-cell interactions in cancer and vascular disease. *J. Biol. Chem.* **2002**, *277*, 4593–6.
4. Wight, T.N.; Merrilees, M.J. Proteoglycans in atherosclerosis and restenosis: Key roles for versican. *Circ. Res.* **2004**, *94*, 1158–1167.
5. Ponting, J.; Howell, A.; Pye, D.; Kumar, S. Prognostic relevance of serum hyaluronan levels in patients with breast cancer. *Int. J. Cancer* **1992**, *52*, 873–6.
6. Ropponen, K.; Tammi, M.; Parkkinen, J.; Eskelinen, M.; Tammi, R.; Lipponen, P.; Agren, U.; Alhava, E.; Kosma, V.M. Tumor cell-associated hyaluronan as an unfavorable prognostic factor in colorectal cancer. *Cancer Res.* **1998**, *58*, 342–7.
7. Chanmee, T.; Ontong, P.; Mochizuki, N.; Kongtawelert, P.; Konno, K.; Itano, N. Excessive Hyaluronan Production Promotes Acquisition of Cancer Stem Cell Signatures through the Coordinated Regulation of Twist and the Transforming Growth Factor β (TGF-β)-Snail Signaling Axis. *J. Biol. Chem.* **2014**, *289*, 26038–26056.
8. Wang, L.; Jia, E. Ovarian cancer targeted hyaluronic acid-based nanoparticle system for paclitaxel delivery to overcome drug resistance. *Drug Deliv.* **2015**, *7544*, 1–8.
9. Yang, X; Iyer, AK; Singh, A; Milane, L; Choy, E; Hornicek, FJ; Amiji, MM; Duan, Z. Cluster of Differentiation 44 Targeted Hyaluronic Acid Based Nanoparticles for MDR1 siRNA Delivery to Overcome Drug Resistance in Ovarian Cancer. *Pharm Res.* **2015**, *32(6)*:2097-109.

Supplementary Figure S3. Reference tissue expression control of scoring guidelines

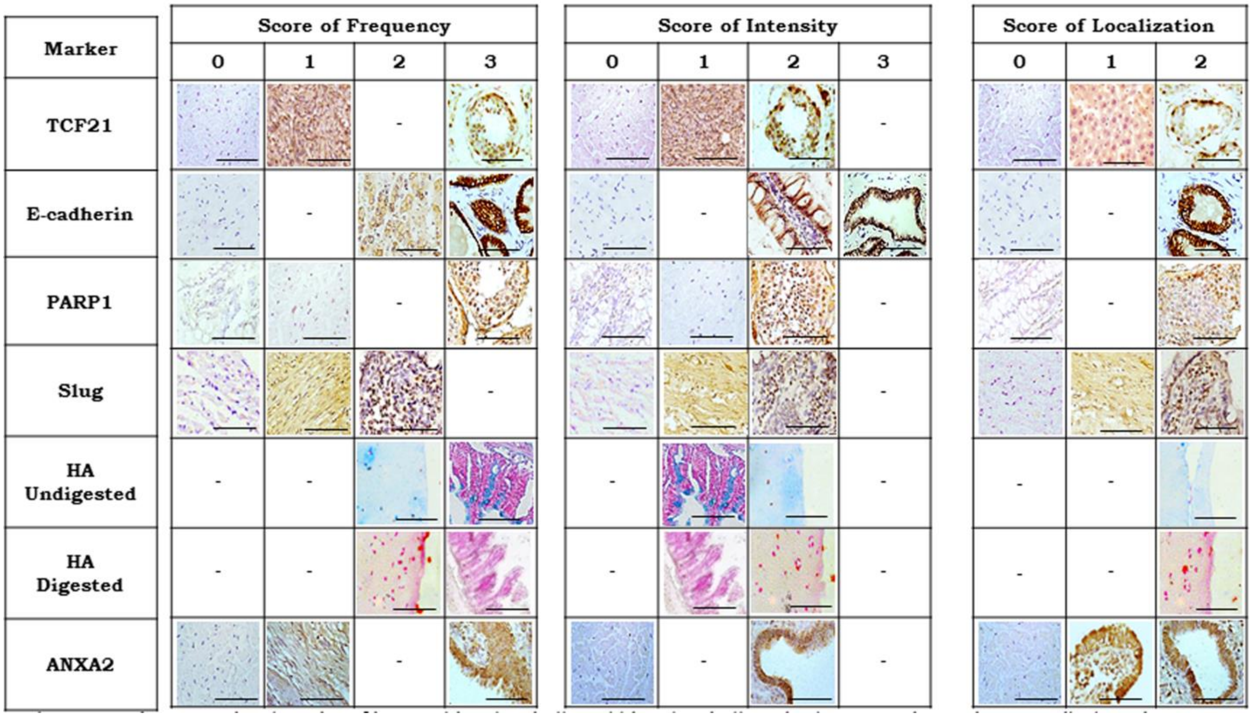

Supplementary Figure S3. Visual scoring of immunohistochemically and histochemically stained FFPE sections using an antibody against TCF21, E-cadherin, PARP1, Slug, ANXA2 and histochemically stained HA in normal human tissues. The marker staining intensities were evaluated by visual scoring for frequency, intensity and localization. TCF21: S_{Freq} – Score 0 (cardiac myocytes), 1 (stromal cells of ovary), 3 (germinal basal cells of testis); S_{Int} – Score 0 (cardiac myocytes), 1 (stromal cells of ovary), 2 (germinal basal cells of testis); S_{Loc} – Score 0 (cardiac myocytes), 1 (hepatocytes of liver), 2 (germinal basal cells of testis). E-cadherin: S_{Freq} – Score 0 (cardiac myocytes), 2 (liver hepatocytes), 3 (epithelial cells of prostate); S_{Int} – Score 0 (cardiac myocytes), 2 (epithelial cells of small intestine), 3 (epithelial cells of prostate); S_{Loc} – Score 0 (cardiac myocytes) and 2 (epithelial cells of prostate). PARP1: S_{Freq}– Score 0 (mucosa of small intestine), 1 (cardiac myocytes), 3 (germinal basal cells of testis); S_{Int} –Score 0 (mucosa of small intestine), 1 (cardiac myocytes), 2 (germinal basal cells of testis); and S_{Loc} – Score 0 (mucosa of small intestine), 2 (germinal basal cells of testis). Slug: S_{Freq} – Score 0 (cardiac myocytes), 1 (smooth muscle of appendix), 2 (lymphocytes of small intestine); S_{Int}–Score 0 (cardiac myocytes), 1 (smooth muscle of appendix), 2 (lymphocytes of small intestine); and S_{Loc} –Score 0 (cardiac myocytes), 1 (somatic muscles of appendix), 2 (lymphocytes of small intestine). HA: S_{Freq} – Score 2 (cartilage), 3 (sub-mucosa of small intestine); S_{Int}– Score 1 (sub-mucosa of small intestine), 2 (cartilage); and S_{Loc} –Score 2 (cartilage). ANXA2: S_{Freq}– Score 0 (cardiac myocytes), 1 (somatic muscle of small intestine), 3 (epithelial cells of gall bladder); S_{Int}– Score 0 (cardiac myocytes), 2 (epithelial cells of gall bladder); and S_{Loc}– Score 0 (cardiac myocytes), 1 (stromal cells of gall bladder), 2 (epithelial cells of gall bladder). Scale bar is 100µm.

Supplementary Figure S4. Representative TMA case for CCM-Class and DP-Class

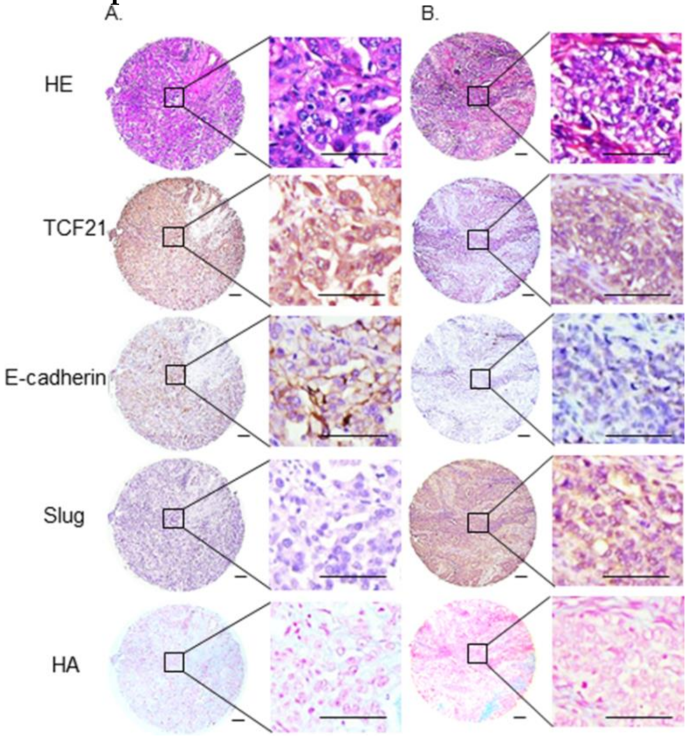

Supplementary Figure S5. Microphotographs of TMA cores stained by HE, IHC for TCF21, E-cadherin, Slug and histochemically by Alcian Blue for Hyaluronan (rows 1, 2, 3, 4, 5 respectively) representative of A. CCM-Class and B. DP-Class. Scale bar is 100μm.

Supplementary Table S2. Biomarker and Class Indices of normal and HGSC cases in TMA leads to Class identification

	Biomarker Index				Class Index	
**TMA cores**	**BI_{TCF21}**	**BI_{CDH1}**	**BI_{Slug}**	**BI_{HA}**	**CI_{CCM}**	**CI_{EMT}**
**A1, B1**	0.61	0.56	0.61	0.78	0.58	0.69
**A2, B2**	0.83	0.19	0.56	0.78	0.51	0.67
**A6, B6**	0.72	0.89	0.00	0.78	0.81	0.39
**C1, D1**	0.72	0.89	0.72	0.78	0.81	0.75
**C2, D2**	0.72	0.61	0.61	0.78	0.67	0.69
**C5, D5**	0.72	0.78	0.00	1.00	0.75	0.5
**C7, D7**	0.72	0.56	0.61	0.72	0.64	0.67
**C8, D8**	0.72	0.61	0.31	0.78	0.67	0.54
**G6, H6**	0.72	0.72	0.72	0.83	0.72	0.78
**G13, H13**	0.72	0.89	0.61	0.78	0.81	0.69
**I3, J3**	0.61	0.72	0.67	0.83	0.67	0.75
**I4, J4**	0.67	0.67	0.67	0.89	0.67	0.78
**I9, J9**	0.67	0.61	0.00	0.78	0.64	0.39
**I11, J11**	0.72	0.44	0.61	0.78	0.58	0.69
**I13, J13**	0.72	0.67	0.61	0.78	0.69	0.69

Normal ovary case core pairs – A1, B1 and A2, B2
HGSC case core pairs – A6, B6; C1, D1; C2, D2; C5, D5; C7, D7; C8, D8; G6, H6; G13, H113; I3, J3; I4, J4; I9, J9; I11, J11 and I13, J13

Supplementary Table S3. Tumor tissues*obtained from different sites in 96 clinical HGSC cases

	**T (1)**	**O (1)**	**A# (2)**	**T- O (2)**	**T - O - FT (3)**	**T - FT (2)**	**A # (1)**	**FT- A # (2)**
**Chemo-naïve**	22	2	1	17	6	1	0	0
**Chemo-treated**	18	1	2	16	0	1	2	1
**Pre- & post-therapy pairs**	3	0	3$	0	0	0	0	0

* Ovarian (T), fallopian tube (FT), omental(O) tumors or ascites (A) were represented by at least one sample from the respective site; $ Tumor tissues were available for ovarian tumors and/or ascites cell block as chemo-naive and chemo-treated pairs; # ascites cell blocks; numbers in brackets indicate tissues from the same patient

Supplementary Table S4. CI scores for chemo-naïve cases in ovarian tumors paired with omental tumor and fallopian tumor, and ovarian tumor collected with ascites leading to Class assignment

Ovarian-Omentum tumor pairs

Case	Tissue ID	Pair	CI_{CCM}	CI_{EMT}	Class
1	B/2981/09	T	0.00	0.26	EMT
		O	0.30	0.30	DP
2	B/548/10	T	0.00	0.80	EMT
		O	0.20	0.83	EMT
3	B/580/10	T	0.22	0.00	CCM
		O	0.00	0.52	EMT
4	B/320/12	T	0.65	0.19	CCM
		O	0.22	0.26	DP
5	B/1029/12	T	0.46	0.26	CCM
		O	0.72	0.52	CCM
6	B/3392/12	T	0.39	0.26	DP
		O	0.44	0.44	DP
7	B/8/13	T	0.26	0.30	DP
		O	0.30	0.30	DP
8	B/343/13	T	0.35	0.39	DP
		O	0.30	0.19	DP
9	B/4/14	T	0.00	0.30	EMT
		O	0.00	0.19	DN
10	B/991/14	T	0.00	0.26	EMT
		O	0.46	0.26	DP
11	B/474/15	T	0.30	0.30	DN
		O	0.33	0.26	DP
12	B/1937/15	T	0.74	0.59	DP
		O	0.63	0.46	DP
13	B/2972/15	T	0.17	0.30	DP
		O	0.00	0.00	DN
14	B/2987/15	T	0.00	0.26	EMT
		O	0.00	0.26	EMT
15	MB/195/12	T	0.00	0.26	EMT
		O	0.00	0.26	EMT
16	1511/14	T	0.52	0.22	CCM
		O	0.00	0.26	EMT
17	1866/13	T	0.00	0.26	CCM
		O	0.20	0.26	DP

Ovarian-Omentum-Fallopian Tube tumors

Case	Tissue ID	Pair	CI_{CCM}	CI_{EMT}	Class
1	B/1716/09	T	0.17	0.30	DP
		O	0.24	0.30	DP
		F	0.00	0.30	EMT
2	B/3136/09	T	0.48	0.30	CCM
		O	0.22	0.00	CCM
		F	0.65	0.26	CCM
3	B/825/10	T	0.26	0.59	EMT
		O	0.59	0.74	DP
		F	0.22	0.26	DN
4	B/2774/12	T	0.33	0.30	DP
		O	0.48	0.30	DP
		F	0.19	0.30	DP
5	B/749/13	T	0.52	0.50	DP
		O	0.57	0.63	DP
		F	0.57	0.30	CCM
6	B/1627/13	T	0.46	0.26	CCM
		O	0.19	0.26	DP
		F	0.19	0.00	DN

Ovary-Fallopian Tube tumor pair

Case	Tissue ID	Pair	CI_{CCM}	CI_{EMT}	Class
1	B/1232/13	T	0.19	0.26	DP
		F	0.26	0.26	DP

Ovary tumor with Ascites

Case	Tissue ID	Pair	CI_{CCM}	CI_{EMT}	Class
1	HT/14/453	T	0.56	0.30	CCM
	HT/14/153	C	0.76	0.26	CCM

Ovarian Tumor (T); Omental Tumor (O)
Cell block (C); Fallopian tube tumor (F)

Supplementary Table S5. CI scores for chemo-naïve cases in unpaired ovarian tumors (n=22) and omental tumors (n=2) leading to Class assignment

Unpaired Ovarian tumor									
**Case**	**Tissue ID**	**CI_{CCM}**	**CI_{EMT}**	**Class**	**Case**	**Tissue ID**	**CI_{CCM}**	**CI_{EMT}**	**Class**
**1**	B/1102/08	0.00	0.22	EMT	**12**	B/1294/11	0.20	0.26	DP
**2**	B/2217/08	0.26	0.00	CCM	**13**	B/1920/11	0.00	0.00	DN
**3**	B/2263/08	0.22	0.26	DP	**14**	B/1338/13	0.26	0.70	EMT
**4**	B/2293/08	0.00	0.00	DN	**15**	B/3091/13	0.26	0.00	CCM
**5**	B/2281/09	0.69	0.64	DP	**16**	B/1781/14	0.00	0.52	EMT
**6**	B/3522/09	0.00	0.44	EMT	**17**	B/1519/15	0.89	0.26	CCM
**7**	B/22/10	0.20	0.48	EMT	**18**	B/2381/15	0.48	0.26	CCM
**8**	B/211/10	0.26	0.44	DP	**19**	B/2283/08	0.00	0.00	DN
**9**	B/799/10	0.00	0.30	EMT	**20**	HT/11/143	0.00	0.00	DN
**10**	B/804/10	0.48	0.30	CCM	**21**	HT/12/1743	0.46	0.52	DP
**11**	B/906/10	0.00	0.26	EMT	**22**	361/13	0.00	0.22	EMT
**Tumor in Omentum**									
**Case**	**Tissue ID**	**CI_{CCM}**	**CI_{EMT}**	**Class**	**Case**	**Tissue ID**	**CI_{CCM}**	**CI_{EMT}**	**Class**
**1**	HT/13/1471	0.20	0.33	DP	**2**	HT/13/4397	0.81	0.26	CCM

Supplementary Table S6. CI scores for chemo-treated cases in unpaired ovarian tumors (n=18) or omental tumor (n=1) or ascites cell block (n=2) leading to Class assignment

Unpaired Ovarian tumors					Unpaired Ovarian tumors				
**Case**	**Tissue ID**	**CI_{CCM}**	**CI_{EMT}**	**Class**	**Case**	**Tissue ID**	**CI_{CCM}**	**CI_{EMT}**	**Class**
1	B/1561/12	0.22	0.22	DP	13	HT/12/34-A7	0.46	0.13	CCM
2	B/914/14	0.00	0.00	DN		HT/12/34-A8	0.35	0.26	DP
3	B/1278/14	0.85	0.20	CCM	14	HT/13/2445	0.00	0.30	EMT
4	B/1582/15	0.78	0.46	CCM	15	HT/12/3871	0.57	0.46	DP
5	B/1481/12	0.54	0.26	CCM	16	1205/14	0.00	0.20	DN
6	OT-20	0.00	0.00	DN	17	1551/14	0.63	0.20	CCM
7	OT-25	0.00	0.30	EMT	18	1268/15	0.46	0.30	DP
8	OT-28	0.00	0.22	EMT	**CT omental tumor**				
9	OT-31	0.00	0.30	EMT	1	1217/14	0.26	0.46	EMT
10	HT/13/5184-B7	0.81	0.30	CCM	**CT ascites cell block**				
	HT/13/5184-A31	0.19	0.44	EMT	1	CT/11/8	0.50	0.26	CCM
11	HT/12/1694-A16	0.50	0.00	CCM	2	CT/12/1510	0.76	0.26	CCM
	HT/12/1694-A11	0.83	0.30	CCM		CT/12/1004	0.43	0.00	CCM
12	HT/12/1491	0.22	0.00	CCM					

Supplementary Table S7. CI scores for chemo-treated cases in ovarian tumors paired with omental tumor (n=16) or ascites (n=2), fallopian tumor (n=1) and FT with ascites leading to Class assignment

Ovarian-Omentum tumor pairs						Ovarian-Omentum tumor pairs					
**Case**	**Tissue ID**	**Site**	**CI_{CCM}**	**CI_{EMT}**	**Class**	**Case**	**Tissue ID**	**Site**	**CI_{CCM}**	**CI_{EMT}**	**Class**
1	B/826/09	T	0.56	0	CCM	12	1968/14	T	0.72	0.19	CCM
	B/827/09	O	0.56	0	CCM			O	0.65	0.3	CCM
2	B/272/11	T	0	0.26	DN	13	1715/14	T	0.63	0.26	CCM
		O	0.3	0.3	DP			O	0.59	0.26	CCM
3	B/2653/12	T	0.72	0.72	DP	14	417/13	T	0.17	0.3	DP
		O	0.48	0.3	DP			O	0	0.26	EMT
4	B/2716/13	T	0.48	0.26	CCM	15	HT/13/4439	T	0.43	0.22	CCM
		O	0.37	0.26	DP		HT/13/4488	O	0.8	0.43	EMT
5	B/1076/14	T	0.69	0.65	DP	16	HT/14/0914-O3	T	0.61	0.26	CCM
		O	0.22	0.72	EMT		HT/14/914-K	O	0.72	0.56	DP
6	B/550/15	T	0.52	0.3	CCM	**Ovarian tumor with ascites**					
		O	0.52	0.3	CCM	1	HT/13/5065	T	0	0.46	EMT
7	MB/952/10	T	0.5	0.26	DP		HT/13/3832	T	0.5	0.3	CCM
		O	0.19	0.3	DP		CT/13/1614	C	0	0	DN
8	MB/443/11	T	0	0.26	EMT	2	HT/12/223-A23	C	0.19	0.43	EMT
		O	0.39	0.46	DP		CT/12/78	T	0.19	0.43	EMT
9	MB/591/11	T	0	0.3	EMT	**Ovarian-Fallopian tube tumors**					
		O	0.17	0.3	DP	1	B/3248/13	T	0	0.26	EMT
10	MB/823/11	T	0.26	0.3	DP			F	0.74	0.31	DN
		O	0.26	0.26	DP	**Fallopian Tube tumors with ascites**					
11	1444/14	T	0.69	0.46	CCM	1	HT/13/3418-A5	F	0.22	0	DN
		O	0	0.3	EMT		CT/13/1087	C	0	0	DN

Supplementary Table S8. CI scores for chemo-naïve and chemo-treated pair cases (n= 6)

Case	Tissue ID	Treatment	CI_{CCM}	CI_{EMT}	Class
1	HT/13/1273	Pre -	0.5	0	CCM
	HT/13/2561-A10	Post -	0.46	0	CCM
2	HT/14/2	Pre -	0.93	0.28	CCM
	HT/14/001890-B9	Post -	0.59	0.3	CCM
	CT/14/591	Post -	0.8	0	CCM
	HT/14/1890-B12	Post -	0.59	0.22	CCM
3	HT/13/2610	Pre -	0.17	0.22	DP
	HT/13/004447-A1	Post -	0.52	0.3	CCM
4	CT/12/1099	Pre -	0.39	0.2	CCM
	HT/12/2879-B2	Post -	0.48	0.2	CCM
	HT/12/2879-A10	Post	0.17	0.17	DN
5	CT/13/1081	Pre -	0.72	0.3	CCM
	HT/12/3173-A4	Post -	0.17	0	DN
	HT/12/3173-B2	Post	0.43	0.3	DP
	CT/12/1236	Post	0	0.22	EMT
6	HT/13/1296	Pre -	0.17	0.26	DP
	CT/12/401	Pre -	0.35	0	CCM
	HT/13/2513-A16	Post	0.39	0	CCM
	HT/13/2513-A20	Post -	0.22	0	CCM
	CT/13/763	Post -	0.48	0	CCM

Supplementary Table S9. Class index scores and assignment to classes for chemo-naïve ovarian tumors paired with fallopian tube tumor and omental tumor (n=6)

	**Ovarian tumor**			**Fallopian tube tumor**			**Omental tumor**		
**Case ID**	**CI_{CCM}**	**CI_{EMT}**	**Class**	**CI_{CCM}**	**CI_{EMT}**	**Class**	**CI_{CCM}**	**CI_{EMT}**	**Class**
B/2774/12	0.33	0.30	DP	0.19	0.3	DP	0.48	0.3	DP
B/3136/09	0.48	0.30	CCM	0.65	0.26	CCM	0.22	0	CCM
B/1627/13	0.46	0.26	CCM	0.19	0.26	DP	0.19	0.26	DP
B/825/10	0.26	0.59	EMT	0.22	0.26	DP	0.59	0.74	DP
B/749/13	0.52	0.50	DP	0.57	0.30	CCM	0.57	0.63	DP
B/1716/09	0.17	0.30	DP	0.00	0.30	EMT	0.24	0.3	DP

Supplementary Table S10. Class comparison of tumors of ovary, fallopian tube and omentum (n=6)

**Class**	**Ovarian tumor**	**Fallopian tube tumor**	**Omental tumor**
CCM	2	2	1
EMT	1	1	0
DP	3	3	5

Supplementary Table S11. Class comparison of tumors of ovary, fallopian tube, omentum and ascites cell block

	Ovarian tumors		Fallopian tube tumors*		Omental tumors*		Ascites cell block*	
	Naïve	Treated	Naïve	Treated	Naïve	Treated	Naïve	Treated
Class	n=50	n=52	n=7	n=2	n=26	n=17	n=4	n=9
CCM	14	25	2	1	5	4	3	5
EMT	15	12	1	0	4	5	0	2
DN	4	5	0	0	2	0	0	2
DP	17	10	4	1	15	8	1	0
Class Comparison CCM *vs.* EMT+DN+DP in ovarian tumors upon chemo-treatment								
Chi square =		3.58						
p value =		0.05						
Class Comparison CCM *vs.* EMT+DN+DP in combined tumor sites upon chemo-treatment								
Chi square =		22.88						
p value =		1.72E-06						

* Class comparison is not applicable as sample size is less than 50

Supplementary Figure S5. Scatter plot of tumors of chemo-naïve and chemo-treated ovary, fallopian tube and omentum tumors, and pre-post tumor pairs

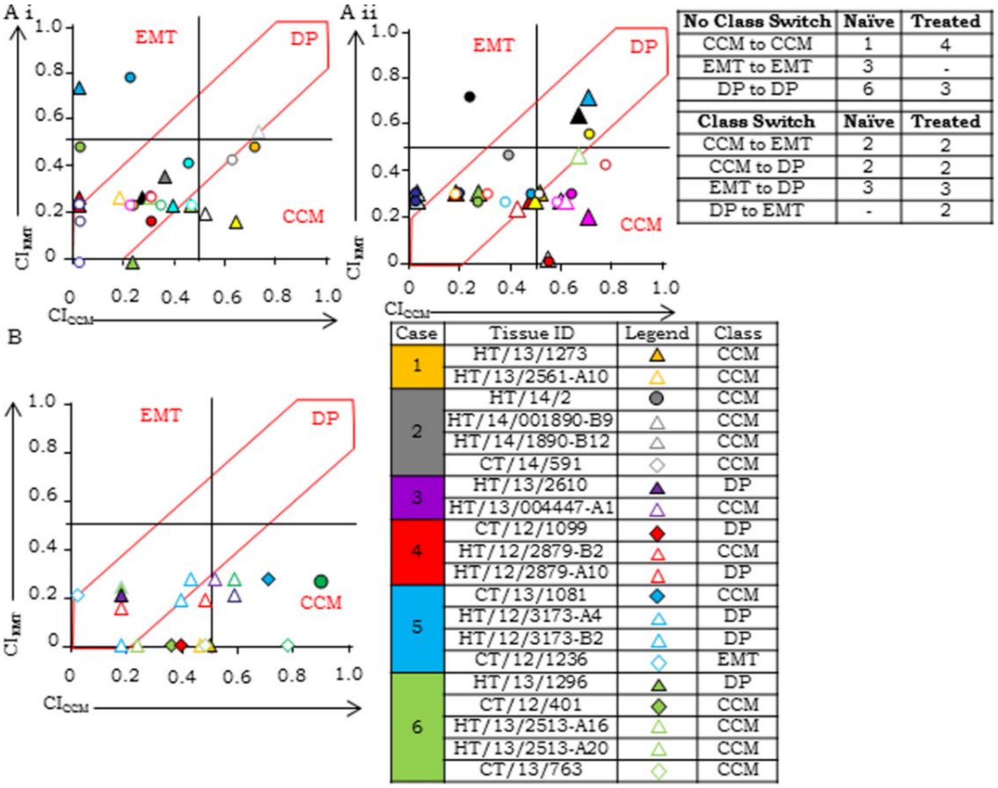

Supplementary Figure S5. Scatter plot of tumors of (A) (i) chemo-naïve (n=17) and (ii) chemo-treated (n=16) ovary, fallopian tube and omentum tumors, and (B) pre-post tumor pairs (n=6)

Supplementary Table S12: Group analysis of tumors of ovary, fallopian tube, omentum and ascites stratified into respective class for chemo-naïve and chemo-treated cases.

	Group 'A'				Group 'C'							
	Chemo-naïve				Chemo-naive				Chemo-treated			
Case	CI_ CCM_T	CI_ CCM_O	CI_ EMT_T	CI_ EMT_O	CI_ CCM_T	CI_ CCM_O	CI_ EMT_T	CI_ EMT_O	CI_ CCM_T	CI_ CCM_O	CI_ EMT_T	CI_ EMT_O
1	0.00	0.30	0.26	0.30	0.33	0.48	0.30	0.30	0.56	0.56	0.00	0.00
2	0.00	0.20	0.80	0.83	0.48	0.22	0.30	0.00	0.00	0.30	0.26	0.30
3	0.22	0.00	0.00	0.52	0.46	0.19	0.26	0.26	0.72	0.48	0.72	0.30
4	0.65	0.22	0.19	0.26	0.26	0.00	0.00	0.26	0.48	0.37	0.26	0.26
5	0.46	0.72	0.26	0.52	0.26	0.59	0.59	0.74	0.69	0.22	0.65	0.72
6	0.39	0.44	0.26	0.44	0.52	0.57	0.50	0.63	0.52	0.52	0.30	0.30
7	0.26	0.30	0.30	0.30	0.17	0.24	0.30	0.30	0.50	0.19	0.26	0.30
8	0.35	0.30	0.39	0.19					0.00	0.39	0.26	0.46
9	0.00	0.00	0.30	0.19					0.00	0.17	0.30	0.30
10	0.00	0.46	0.26	0.26					0.26	0.26	0.30	0.26
11	0.30	0.33	0.30	0.26					0.69	0.00	0.46	0.30
12	0.74	0.63	0.59	0.46					0.72	0.65	0.19	0.30
13	0.17	0.00	0.30	0.00					0.63	0.59	0.26	0.26
14	0.00	0.00	0.26	0.26					0.17	0.00	0.30	0.26
15	0.00	0.00	0.26	0.26					0.43	0.80	0.22	0.43
16	0.52	0.00	0.22	0.26					0.61	0.72	0.26	0.56
17	0.00	0.20	0.26	0.26								

ANOVA					
	Sum of Squares	df	Mean Square	F	Sig.
Between Groups	.525	11	.048	1.072	.387
Within Groups	6.582	148	.044		
Total	7.107	159			

*. The mean difference is significant at the 0.05 level and highlighted with yellow background.

The variables 1-12 are Chemo-Naïve tumor (T of TO pair)-CI_{CCM};Chemo-Naïve tumor (O of TO pair)-CI_{CCM}; Chemo-Naïve tumor (T of TO pair)_CI_{EMT};Chemo-Naïve tumor (O of TO pair)_CI_{EMT}; Chemo-Naïve tumor (T of TFO)_CI_{CCM};Chemo-Naïve tumor (O of TFO)_CI_{CCM};Chemo-Naïve tumor (T of TFO)_CI_{EMT};Chemo-Naïve tumor (O of TFO)_CI_{EMT};Chemo-treated tumor (T of TO pair)_CI_{CCM}; Chemo-treated tumor (O of TO pair)_CI_{CCM};Chemo-treated tumor (T of TO pair)_CI_{EMT};and Chemo-treated tumor (O of TO pair)_CI_{EMT}.

Supplementary Figure S6. Effect of marker expression with Stage of HGSC at presentation

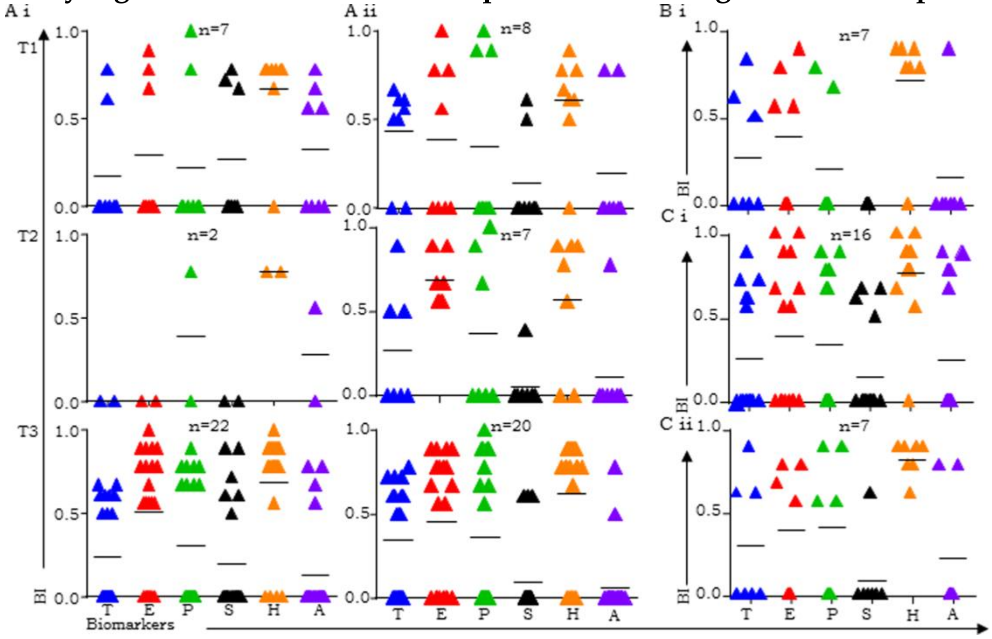

Supplementary Figure S6. Expression of BI for TCF21 (T), E-cadherin (E), PARP1 (P), Slug (S), Hyaluronan (H) and ANXA2 (A) in A. Ovarian tumors i. chemo-naïve and ii. chemo-treated for stages T1, T2 and T3; B. Fallopian tube chemo-naïve for stage T3; C. Omental tumors for stage T3 i. chemo-naïve and ii. chemo-treated.

References

1. Arab, K.; Smith, L.T.; Gast, A.; Weichenhan, D.; Huang, J.P.-H.; Claus, R.; Hielscher, T.; Espinosa, A.V; Ringel, M.D.; Morrison, C.D.; et al. Epigenetic deregulation of TCF21 inhibits metastasis suppressor KISS1 in metastatic melanoma. *Carcinogenesis* **2011**, *32*, 1467–1473.
2. Costa, V.L.; Henrique, R.; Danielsen, S.A.; Eknaes, M.; Patrício, P.; Morais, A.; Oliveira, J.; Lothe, R.A.; Teixeira, M.R.; Lind, G.E.; et al. TCF21 and PCDH17 methylation: An innovative panel of biomarkers for a simultaneous detection of urological cancers. *Epigenetics* **2011**, *6*, 1120–1130.
3. Dabbs, D.J. *Diagnostic Immunohistochemistry*, 4th Edition: Elsevier; 2013.
4. Dai, Y.; Duan, H.; Duan, C.; Zhou, R.; He, Y.; Tu, Q.; Shen, L. Down-regulation of TCF21 by hypermethylation induces cell proliferation, migration and invasion in colorectal cancer. *Biochem. Biophys. Res. Commun.* **2015**, *469*, 430–436.
5. Richards, K.L.; Zhang, B.; Sun, M.; Dong, W.; Churchill, J.; Bachinski, L.L.; Wilson, C.D.; Baggerly, K.A.; Yin, G.; Hayes, D.N.; et al. Methylation of the candidate biomarker TCF21 is very frequent across a spectrum of early-stage nonsmall cell lung cancers. *Cancer* **2011**, *117*, 606–617.
6. Tan, J.; Zhang, G.; Liu, R.; Zhou, M.; Li, Z.; Wu, Z. Over-expression of transcription factor 21 inhibits the proliferation and migration and promotes apoptosis of SMMC-7721 cells. *Xi Bao Yu Fen Zi Mian Yi Xue Za Zhi* **2015**, *31*, 884–888.
7. Tessema, M.; Willink, R.; Do, K.; Yu, Y.Y.; Yu, W.; Machida, E.O.; Brock, M.; Van Neste, L.; Stidley, C.A.; Baylin, S.B.; et al. Promoter methylation of genes in and around the candidate lung cancer susceptibility locus 6q23-25. *Cancer Res.* **2008**, *68*, 1707–1714.
8. Wang, J.; Gao, X.; Wang, M.; Zhang, J. Clinicopathological significance and biological role of TCF21 mRNA in breast cancer. *Tumour Biol.* **2015**, *36*, 8679–8683.
9. Weiss, D.; Stockmann, C.; Schrödter, K.; Rudack, C. Protein expression and promoter methylation of the candidate biomarker TCF21 in head and neck squamous cell carcinoma. *Cell. Oncol. (Dordr).* **2013**, *36*, 213–224.
10. Bačić, B.; Haller, H.; Mrklić, I.; Košta, V.; Čarić, A.; Tomić, S. Prognostic role of E-cadherin in patients with advanced serous ovarian cancer. *Arch. Gynecol. Obstet.* **2013**, *287*, 1219–1224.
11. Batlle, E.; Sancho, E.; Francí, C.; Domínguez, D.; Monfar, M.; Baulida, J.; García De Herreros, A. The transcription factor snail is a repressor of E-cadherin gene expression in epithelial tumour cells. *Nat. Cell Biol.* **2000**, *2*, 84–89.
12. Berx, G.; van Roy, F. Involvement of Members of the Cadherin Superfamily in Cancer. *Cold Spring Harb. Perspect. Biol.* **2009**, *1*, a003129–a003129.
13. Bolós, V.; Peinado, H.; Pérez-Moreno, M.A.; Fraga, M.F.; Esteller, M.; Cano, A. The transcription factor Slug represses E-cadherin expression and induces epithelial to mesenchymal transitions: a comparison with Snail and E47 repressors. *J. Cell Sci.* **2003**, *116*, 499–511.
14. Cano, A.; Pérez-Moreno, M.A.; Rodrigo, I.; Locascio, A.; Blanco, M.J.; del Barrio, M.G.; Portillo, F.; Nieto, M.A. The transcription factor snail controls epithelial-mesenchymal transitions by repressing E-cadherin expression. *Nat. Cell Biol.* **2000**, *2*, 76–83.
15. De Craene, B.; Berx, G. Regulatory networks defining EMT during cancer initiation and progression. *Nat. Rev. Cancer* **2013**, *13*, 97–110.
16. Wang, Y.-P.; Wang, M.-Z.; Luo, Y.-R.; Shen, Y.; Wei, Z.-X. Lentivirus-mediated shRNA interference targeting SLUG inhibits lung cancer growth and metastasis. *Asian Pac. J. Cancer Prev.* **2012**, *13*, 4947–51.
17. Rodriguez, F.J.; Lewis-Tuffin, L.J.; Anastasiadis, P.Z. E-cadherin's dark side: possible role in tumor progression. *Biochim. Biophys. Acta* **2012**, *1826*, 23–31.
18. Wong, A.S.T.; Gumbiner, B.M. Adhesion-independent mechanism for suppression of tumor cell invasion by E-cadherin. *J. Cell Biol.* **2003**, *161*, 1191–1203.
19. Chow, J.P.H.; Man, W.Y.; Mao, M.; Chen, H.; Cheung, F.; Nicholls, J.; Tsao, S.W.; Li Lung, M.; Poon, R.Y.C. PARP1 is overexpressed in nasopharyngeal carcinoma and its inhibition enhances radiotherapy. *Mol. Cancer Ther.* **2013**, *12*, 2517–2528.

20. Iglehart, J.D.; Silver, D.P. Synthetic Lethality-A New Direction in Cancer-Drug Development. *N. Engl. J. Med.* **2009**, *361*, 189–191.
21. Iyama, T.; Wilson, D.M. DNA repair mechanisms in dividing and non-dividing cells. *DNA Repair (Amst).* **2013**, *12*, 620–636.
22. Kummar, S.; Chen, A.; Parchment, R.E.; Kinders, R.J.; Ji, J.; Tomaszewski, J.E.; Doroshow, J.H. Advances in using PARP inhibitors to treat cancer. *BMC Med.* **2012**, *10*, 25.
23. Magdalena, K.; Monika, Z.; Adam, G.; Magdalena, R.; Marzena, L.; Wojciech, B.; Janusz, L.; Bartosz, W. Detection of somatic BRCA1/2 mutations in ovarian cancer - next-generation sequencing analysis of 100 cases. *Cancer Med.* **2016**, *5*, 1640–1646.
24. Marques, M.; Beauchamp, M.-C.; Fleury, H.; Laskov, I.; Qiang, S.; Pelmus, M.; Provencher, D.; Mes-Masson, A.-M.; Gotlieb, W.H.; Witcher, M. Chemotherapy reduces PARP1 in cancers of the ovary: implications for future clinical trials involving PARP inhibitors. *BMC Med.* **2015**, *13*, 217.
25. Ossovskaya, V.; Koo, I.C.; Kaldjian, E.P.; Alvares, C.; Sherman, B.M. Upregulation of Poly (ADP-Ribose) Polymerase-1 (PARP1) in Triple-Negative Breast Cancer and Other Primary Human Tumor Types. *Genes Cancer* **2010**, *1*, 812–821.
26. Castro Alves, C.; Rosivatz, E.; Schott, C.; Hollweck, R.; Becker, I.; Sarbia, M.; Carneiro, F.; Becker, K.-F. Slug is overexpressed in gastric carcinomas and may act synergistically with SIP1 and Snail in the down-regulation of E-cadherin. *J. Pathol.* **2007**, *211*, 507–515.
27. Ding, G.; Feng, C.; Jiang, H.; Ding, Q.; Zhang, L.; Na, R.; Xu, H.; Liu, J. Combination of rapamycin, CI-1040, and 17-AAG inhibits metastatic capacity of prostate cancer via Slug inhibition. *PLoS One* **2013**, *8*, e77400.
28. Elloul, S.; Elstrand, M.B.; Nesland, J.M.; Tropé, C.G.; Kvalheim, G.; Goldberg, I.; Reich, R.; Davidson, B. Snail, Slug, and Smad-interacting protein 1 as novel parameters of disease aggressiveness in metastatic ovarian and breast carcinoma. *Cancer* **2005**, *103*, 1631–1643.
29. Giannelli, G.; Bergamini, C.; Fransvea, E.; Sgarra, C.; Antonaci, S. Laminin-5 with transforming growth factor-beta1 induces epithelial to mesenchymal transition in hepatocellular carcinoma. *Gastroenterology* **2005**, *129*, 1375–1383.
30. Kurrey, N.K.; Jalgaonkar, S.P.; Joglekar, A.V; Ghanate, A.D.; Chaskar, P.D.; Doiphode, R.Y.; Bapat, S.A. Snail and slug mediate radioresistance and chemoresistance by antagonizing p53-mediated apoptosis and acquiring a stem-like phenotype in ovarian cancer cells. *Stem Cells* **2009**, *27*, 2059–2068.
31. Kurrey, N.K.; Amit, K.; Bapat, S.A. Snail and Slug are major determinants of ovarian cancer invasiveness at the transcription level. *Gynecol. Oncol.* **2005**, *97*, 155–165.
32. Qian, J.; Liu, H.; Chen, W.; Wen, K.; Lu, W.; Huang, C.; Fu, Z. Knockdown of Slug by RNAi inhibits the proliferation and invasion of HCT116 colorectal cancer cells. *Mol. Med. Rep.* **2013**, *8*, 1055–1059.
33. Riemenschnitter, C.; Teleki, I.; Tischler, V.; Guo, W.; Varga, Z. Stability and prognostic value of Slug, Sox9 and Sox10 expression in breast cancers treated with neoadjuvant chemotherapy. *Springerplus* **2013**, *2*, 695.
34. Shih, J.-Y.; Yang, P.-C. The EMT regulator slug and lung carcinogenesis. *Carcinogenesis* **2011**, *32*, 1299–1304.
35. Shioiri, M.; Shida, T.; Koda, K.; Oda, K.; Seike, K.; Nishimura, M.; Takano, S.; Miyazaki, M. Slug expression is an independent prognostic parameter for poor survival in colorectal carcinoma patients. *Br. J. Cancer* **2006**, *94*, 1816–1822.
36. Uygur, B.; Wu, W.-S. SLUG promotes prostate cancer cell migration and invasion via CXCR4/CXCL12 axis. *Mol. Cancer* **2011**, *10*, 139.
37. Yang, H.W.; Menon, L.G.; Black, P.M.; Carroll, R.S.; Johnson, M.D. SNAI2/Slug promotes growth and invasion in human gliomas. *BMC Cancer* **2010**, *10*, 301.
38. Chen, C.Y.; Lin, Y.S.; Chen, C.L.; Chao, P.Z.; Chiou, J.F.; Kuo, C.C.; Lee, F.P.; Lin, Y.F.; Sung, Y.H.; Lin, Y.T.; et al. Targeting annexin A2 reduces tumorigenesis and therapeutic resistance of nasopharyngeal carcinoma. *Oncotarget* **2015**, *6* (29): 26946–26959. doi: 10.18632/oncotarget.4521
39. Díaz, V.M.; Hurtado, M.; Thomson, T.M.; Reventós, J.; Paciucci, R. Specific interaction of tissue-type plasminogen activator (t-PA) with annexin II on the membrane of pancreatic cancer cells activates plasminogen and promotes invasion in vitro. *Gut* **2004**, *53*, 993–1000.

40. Hajjar, K.A.; Guevara, C.A.; Lev, E.; Dowling, K.; Chacko, J. Interaction of the Fibrinolytic Receptor, Annexin II, with the endothelial cell surface. *J Biol Chem* **1996**, *271*, 21652–21659.
41. Inokuchi, J.; Narula, N.; Yee, D.S.; Skarecky, D.W.; Lau, A.; Ornstein, D.K.; Tyson, D.R. Annexin A2 positively contributes to the malignant phenotype and secretion of IL-6 in DU145 prostate cancer cells. *Int. J. Cancer* **2009**, *124*, 68–74.
42. Mai, J.; Waisman, D.M.; Sloane, B.F. Cell surface complex of cathepsin B/annexin II tetramer in malignant progression. *Biochim. Biophys. Acta* **2000**, *1477*, 215–230.
43. Mohammad, H.S.; Kurokohchi, K.; Yoneyama, H.; Tokuda, M.; Morishita, A.; Jian, G.; Shi, L.; Murota, M.; Tani, J.; Kato, K.; et al. Annexin A2 expression and phosphorylation are up-regulated in hepatocellular carcinoma. *Int. J. Oncol.* **2008**, *33*, 1157–1163.
44. Deng, Y.; Chen, C.; Hua, M.; Xi, Q; Liu, R, Yang, S; Liu, j; Zhong, j; Tang, M; Lu, S; Tang, C; Wang, Y. Annexin A2 plays a critical role in epithelial ovarian cancer. *Arch. Gynecol. Obstet.* **2014**, *292*, 175–182.
45. Sharma, M.R.; Koltowski, L.; Ownbey, R.T.; Tuszynski, G.P.; Sharma, M.C. Angiogenesis-associated protein annexin II in breast cancer: Selective expression in invasive breast cancer and contribution to tumor invasion and progression. *Exp. Mol. Pathol.* **2006**, *81*, 146–156.
46. Shiozawa, Y.; Havens, A.M.; Jung, Y.; Ziegler, A.M.; Pedersen, E.A.; Wang, J.; Wang, J.; Lu, G.; Roodman, G.D.; Loberg, R.D.; et al. Annexin II/annexin II receptor axis regulates adhesion, migration, homing, and growth of prostate cancer. *J. Cell. Biochem.* **2008**, *105*, 370–380.
47. Vishwanatha, J.K.; Chiang, Y.; Kumble, K.D.; Hollingsworth, M.A.; Pour, P.M. Enhanced expression of annexin II in human pancreatic carcinoma cells and primary pancreatic cancers. *Carcinogenesis* **1993**, *14*, 2575–2579.
48. Chanmee, T.; Ontong, P.; Mochizuki, N.; Kongtawelert, P.; Konno, K.; Itano, N. Excessive Hyaluronan Production Promotes Acquisition of Cancer Stem Cell Signatures through the Coordinated Regulation of Twist and the Transforming Growth Factor β (TGF-β)-Snail Signaling Axis. *J. Biol. Chem.* **2014**, *289*, 26038–26056.
49. Platt, V.M.; Szoka, F.C. Anticancer therapeutics: targeting macromolecules and nanocarriers to hyaluronan or CD44, a hyaluronan receptor. *Mol. Pharm.* **2008**, *5*, 474–486.
50. Ponting, J.; Howell, A.; Pye, D.; Kumar, S. Prognostic relevance of serum hyaluronan levels in patients with breast cancer. *Int. J. Cancer* **1992**, *52*, 873–876.
51. Ropponen, K.; Tammi, M.; Parkkinen, J.; Eskelinen, M.; Tammi, R.; Lipponen, P.; Agren, U.; Alhava, E.; Kosma, V.M. Tumor cell-associated hyaluronan as an unfavorable prognostic factor in colorectal cancer. *Cancer Res.* **1998**, *58*, 342–347.
52. Toole, B.P. Hyaluronan: from extracellular glue to pericellular cue. *Nat. Rev. Cancer* **2004**, *4*, 528–539.
53. Wang, L.; Jia, E. Ovarian cancer targeted hyaluronic acid-based nanoparticle system for paclitaxel delivery to overcome drug resistance. *Drug Deliv.* **2015**, *7544*, 1–8.
54. Weigel, P.H.; DeAngelis, P.L. Hyaluronan synthases: a decade-plus of novel glycosyltransferases. *J. Biol. Chem.* **2007**, *282*, 36777–36781.
55. Wight, T.N.; Merrilees, M.J. Proteoglycans in atherosclerosis and restenosis: Key roles for versican. *Circ. Res.* **2004**, *94*, 1158–1167.
56. Yang, X.; Iyer, A.K.; Singh, A.; Milane, L.; Choy, E.; Hornicek, F.J.; Amiji, M.M.; Duan, Z. Cluster of Differentiation 44 Targeted Hyaluronic Acid Based Nanoparticles for MDR1 siRNA Delivery to Overcome Drug Resistance in Ovarian Cancer. *Pharm. Res.* **2014**, *32*, 2097–2109.
